# Development of an ex-vivo porcine lower urinary tract model to evaluate the performance of urinary catheters

**DOI:** 10.1038/s41598-022-21122-6

**Published:** 2022-10-24

**Authors:** Fabio Tentor, Brit Grønholt Schrøder, Simon Nielsen, Lars Schertiger, Kristian Stærk, Thomas Emil Andersen, Per Bagi, Lene Feldskov Nielsen

**Affiliations:** 1 Coloplast A/S, Holtedam 1, 3050 Humlebæk, Denmark; 2 Research Unit of Clinical Microbiology, University of Southern Denmark, J.B. Winsløws Vej 21, 5000 Odense, Denmark; 3 Department of Clinical Microbiology, Odense University Hospital, J.B. Winsløws Vej 21, 5000 Odense, Denmark; 4 Department of Urology, Centre for Cancer and Organ Diseases, Rigshospitalet, Blegdamsvej 9, 2100 København, Denmark

**Keywords:** Urinary tract, Bladder, Ureter, Urethra, Bladder, Ureter, Urethra, Health care, Quality of life, Experimental models of disease, Medical research, Preclinical research, Engineering, Biomedical engineering, Urology, Urological manifestations

## Abstract

Intermittent catheterization is the gold standard method for bladder management in individuals with urinary retention and/or incontinence. It is therefore important to understand the performance of urinary catheters, especially on parameters associated to risks of developing urinary tract infections, and that may impact the quality of life for urinary catheter users. Examples of such parameters include, urine flowrate, occurrence of flow-stops, and residual urine left in the bladder after flow-stop. Reliable in-vitro and/or ex-vivo laboratory models represent a strong asset to assess the performance of urinary catheters, preceding and guiding in-vivo animal studies and/or human clinical studies. Existing laboratory models are generally simplified, covering only portions of the catheterization process, or poorly reflect clinical procedures. In this work, we developed an ex-vivo porcine lower urinary tract model that better reflects the catheterization procedure in humans and allows to investigate the performance of standard of care catheters. The performance of three standard of care catheters was investigated in the developed model showing significant differences in terms of flowrate. No differences were detected in terms of residual volume in the bladder at first flow-stop also when tuning the abdominal pressure to mimic a sitting down and standing up position. A newly discovered phenomenon named hammering was detected and measured. Lastly, mucosal suction was observed and measured in all standard of care catheters, raising the concern for microtrauma during catheterization and a need for new and improved urinary catheter designs. Results obtained with the ex-vivo model were compared to in-vivo studies, highlighting similar concerns.

## Introduction

Lower urinary tract dysfunctions are common among the population, with prevalence increasing with age^1,2^. Losing control over one's bladder has several implications including a negative effect on the mental wellbeing and general quality of life of the patient^3^. For people with urinary bladder complications, bladder emptying performed by intermittent catheterization (IC) or by the usage of indwelling catheters is the preferred method, allowing them to regain control of their life^4–6^. Intermittent catheters are widely used, requiring 4 to 6 catheterizations per day^7,8^, which make their performance of crucial importance. Factors such as the force needed to insert a catheter in the urethra, friction during insertion, catheter handling, flowrate, flow-stops, mucosal suctions, residual urine, and the need of repositioning, all may have an impact on the performance as well as the patient´s experience during IC. Moreover, residual urine and microtraumas to the urethra or to the bladder, constitute risks for development of urinary tract infections (UTIs)^9^. Understanding each individual catheterization steps can help optimizing the performance of urinary catheters. This increases the need for laboratory models that recapitulate, as close as possible, the conditions urinary catheters are exposed to during use.

Testing of urinary catheters can be performed by mechanical tests according to ISO standards^10^, by classical microbiological tests^11^, or by in-vitro and in-vivo studies. Some in-vitro studies, such as the one developed by Humphreys et al., aim at bridging the gap between pure mechanical testing of friction, and the biological relevance of trauma towards the tissue^12^.

In-vivo studies have also been used to investigate urinary catheters performance. For instance, Smarick et al. performed an animal study on dogs to evaluate the incidence of catheter-associated UTIs^13^. Mandakhalikar et al., used in-vivo animal studies in mice to evaluate the benefits of using antifouling coatings to reduce bacterial colonization in the bladder^14^.

However, porcine models are preferred due to their anatomy and physiology being more relevant for translational medicine towards humans^15,16^. Accordingly, Nielsen et al. developed an in-vivo cystitis porcine model to understand UTIs pathogenesis^17,18^. An in-vivo porcine model was also adapted to facilitate UTIs with indwelling catheters^19^. Overall, these studies isolate and investigate specific properties in detail but fail in describing the overall catheter performance.

Ex-vivo models have been developed to investigate the performance of catheters in conditions that can better simulate the physiological environment of human beings in comparison to in-vitro and mechanical testes while being less expensive and faster than in-vivo studies and clinical trials For example, an ex-vivo porcine bladder model was developed by Parson et al. to perform physiological investigations^20^. Glahn et al., on the other hand, developed a test which makes usage of a porcine bladder to perform hydro-physical analyses and understand what happens when tissue approaches urinary catheters eyelets^21^. Another ex-vivo model was developed to evaluate biofilm colonization^22^. Overall, the currently available ex-vivo models, although highly relevant, make limited use of their full potential and limit their usage on a small portion of the whole IC procedure.

In this study, we developed an ex-vivo porcine lower urinary tract (LUT) model which allows for the entire IC procedure to be evaluated. The endpoints of testing catheter performance with the ex-vivo porcine LUT model can be divided into qualitative- and quantitative-endpoints. Qualitative endpoints include handling of the catheter, occurrence of flow-stops, presence of mucosal suction, and visual investigations by means of an endoscope. Quantitative endpoints include flowrate, and residual volume at flow-stop. Furthermore, the ex-vivo porcine LUT model can be coupled with pressure sensors to allow for the measurement of the pressure within the catheter during, for example mucosal suction.

An additional advantage of the developed ex-vivo porcine LUT model, in comparison to other ex-vivo models, is the possibility of adjusting the pressure applied to the bladder, this is essential to mimic the abdominal pressure present in humans, which vary greatly depending on the patient position^23,24^. In this work, two abdominal pressures were used to represent a sitting down and a standing up position during IC.

The performance of intermittent standard of care (SOC) catheters (i.e., conventional 2-eyelet catheters of well-known brands) was evaluated in this study focusing on four performance parameters:

1. *Flowrate:* since IC users must catheterize several times per day, the amount of time necessary for this procedure is relevant.
2. *Residual volume at first flow-stops:* if an IC user withdraws the urinary catheter as soon as flow stops, failure in completely emptying the urinary bladder can occur. As residual urine has been previously listed as one of the risk factors for developing UTIs, this parameter requires special attention^9^.
3. *Mucosal suction:* event previously reported in the literature^21,25^, originating from sudden flow-stop. When mucosal suction happens, because of the negative pressure originating from the water column in the catheter, tissue of the bladder mucosa is pulled inside of the catheter lumen in an abrupt and vigorous manner. The event raises concerns for potential microtrauma to the bladder mucosa, traumas that have been previously listed as one of the UTIs risk factors^9^ and may be associated to discomfort or pain while performing IC.
4. *Hammering:* this phenomenon was discovered while testing in the ex-vivo porcine LUT model and confirmed in the in-vivo animal studies. Consisting of a quick succession of mucosal suctions without the simultaneous presence of a flow-stop, hammering can be perceived by the operator holding the catheter, the tactile sensation corresponds to a vibration/quick pulsating of the catheter. This phenomenon has not been completely understood yet; nor are its implications and clinical relevance clear. Hammering was quantified in this work by means of a pressure sensor inserted directly inside the catheter lumen during catheterization.

Findings from the ex-vivo porcine LUT model, including mucosal suction, pressure fluctuations, and hammering were confirmed in in-vivo animal studies in pigs.

The extensive correlation between the ex-vivo porcine LUT model and the in-vivo animal studies shown in this study, together with the possibility of examining the overall performance of different SOC catheters during IC, as well as the option of varying the abdominal pressure on demand makes the ex-vivo porcine LUT model robust, and better at predicting the performance of catheters in humans. The findings, described throughout the article, raise some concerns over the potential occurrence of microtraumas during IC and questions whether improved, microtrauma-preventive catheter designs, can be developed to improve users´ life quality.

## Materials and methods

### Preparation of the porcine lower urinary tracts

**Figure 1:**
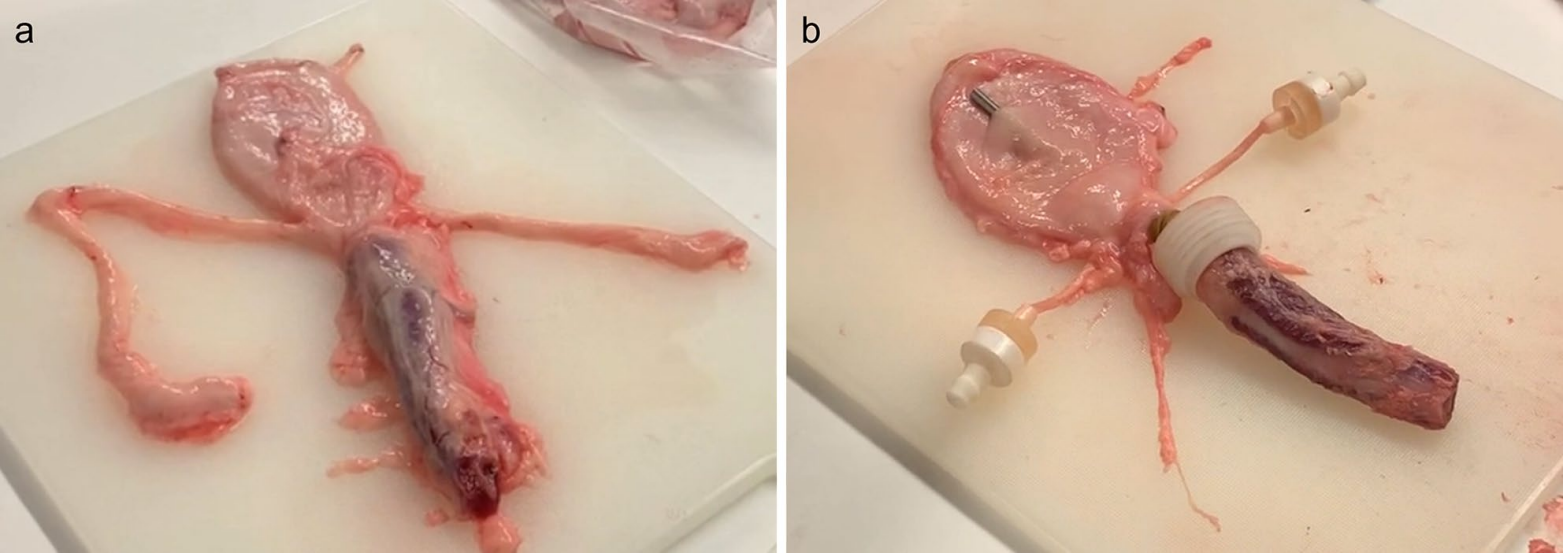
Example of a freshly retrieved male porcine LUT (**a**), and of a male porcine LUT prepared after trimming off the excess fat and placing of the fittings (**b**). In this specific example, a needle was punched through the bladder wall to access the bladder lumen (**b**); this additional access point was never used in the tests described in this manuscript.

Porcine lower urinary tracts (LUTs) were obtained daily from a local slaughterhouse (Glumsø Slagtehus ApS, Glumsø, Denmark) and used fresh upon arrival. Only the male porcine LUTs which bladders were not overextended, that contained a maximum of 250 mL, and the urethra and at least one ureter intact were used (Fig. 1a). After selection, excess fat around the urethra was removed, using a scalpel, exposing the muscular tissue. Similarly, the excess fat surrounding the ureters was also discarded to obtain smooth ureters. Fittings were then placed at the urethra and ureters, necessary to firmly hold the porcine LUT within the model (Fig. 1b). A rubber band was used to mimic the bladder sphincter. When necessary, the bladder wall was punctured with a needle to allow for endoscope access. Lastly, the urethra was measured and cut to the desired length. The developed porcine LUT model could unfortunately not be used with female ex-vivo porcine LUTs as major changes in the fittings to support the urethra and hold it in place were needed and could not be achieved.

### Ex-vivo porcine LUT model set up

**Figure 2:**
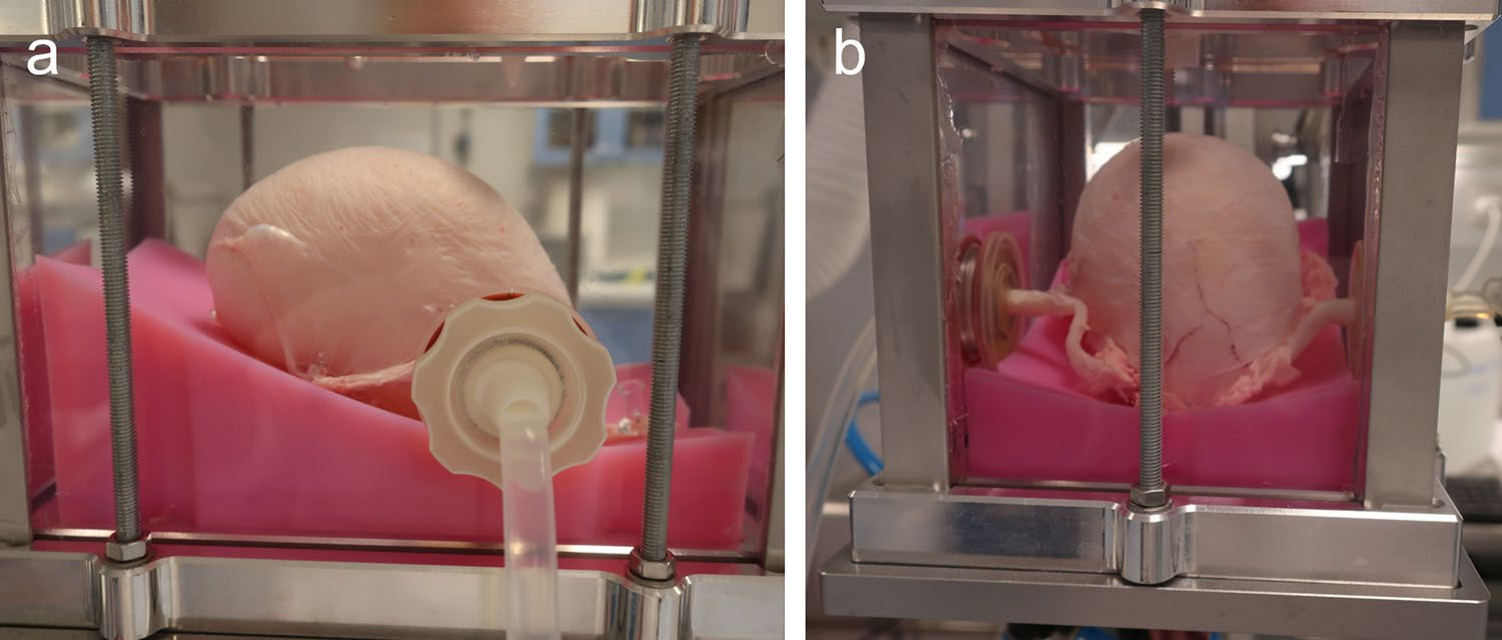
Example of a filled male porcine LUT inside the pressurized tank. The porcine LUT is pinned to the silicone mold (in pink) and connected to the tank via the urethral and ureteral fittings. (**a**) lateral view, (**b**) frontal view.

Once the male porcine LUT was ready, it was placed over a silicone mold that supports the bladders and mimics the pelvic floor. A hole in the silicone mold was used to pass the porcine urethra through. Excess tissue on the bladder was used to pin the bladder to the silicone mold. This was necessary to prevent the lifting of the bladder during its filling. The silicone mold and the male porcine LUT were then inserted into a custom-made tank. The urethral fitting was secured to the bottom, while the ureteral fittings were secured to the side walls. One of the two ureters’ fittings was then connected to a peristaltic pump whereas the other one, if present, was either sealed or used to add specific liquids or material during testing. Figure 2 shows a porcine LUT pinned to the silicone mold, inside the tank and connected by the urethral and ureteral fittings.

**Figure 3:**
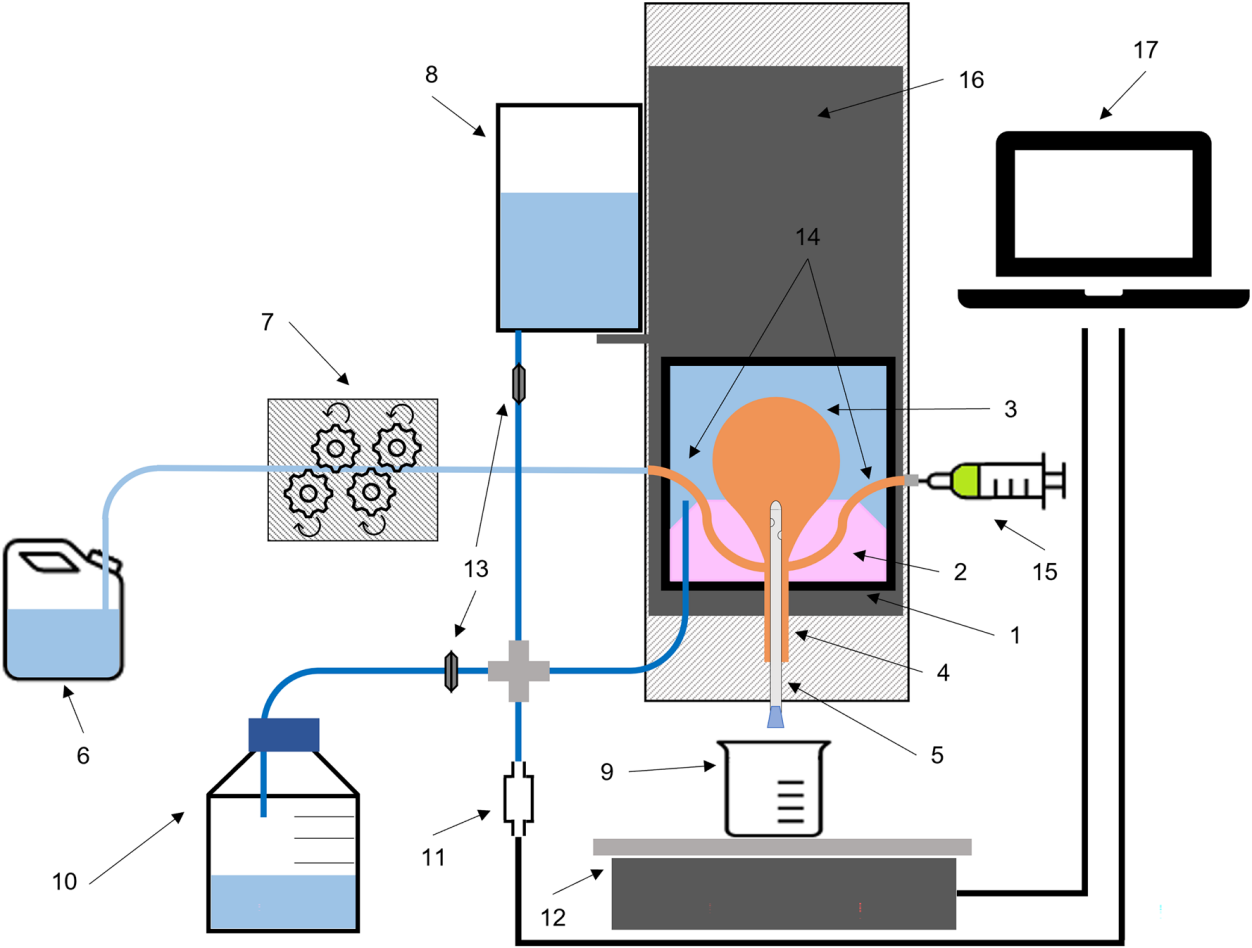
Schematic representation of the ex-vivo porcine LUT model setup. (1) Pressurized tank, (2) silicone mold to mimic the pelvic floor, (3) porcine LUT, (4) porcine urethra, (5) catheter inserted in the bladder through the urethra, (6) reservoir used to fill the bladder through one of the two ureters, (7) peristaltic pump, (8) adjustable water column to apply the desired abdominal pressure in the tank “1”, (9) vessel to collect the volume emptied from the bladder, (10) waste, (11) pressure sensor, (12) weighing scale, (13) ON/OFF valve, (14) ureters, (15) syringe used to add additional solutions/suspensions in the bladder through the second available ureter (can also be used as an access point for an endoscope), (16) LUT model support allowing for the vertical movement of both the tank “1” and the water column “8”, (17) computer collecting data from the pressure sensor “11” and the weighing scale “12”.

The tank was filled with a saline solution of NaCl 150 mM (Sigma Aldrich, St. Louis, US), sealed, and placed under pressure at levels representing the abdominal pressure. The abdominal pressure was applied by a column of saline water connected to the tank. The column height could be adjusted to reach the desired abdominal pressure. For most tests, an abdominal pressure of 50 cmH_2_O (i.e., ≈ 49.0 mbar) was deployed, representing the higher range for abdominal pressures in adult humans in a standing position^23^. The pressure within the tank was monitored through a pressure sensor positioned at a lower point from the tank. Figure 3 presents a schematic representation of the ex-vivo porcine LUT model.

Once the setup was ready, the bladder was filled with 205 ± 5 mL of 150 mM NaCl at a flowrate of 5.5 mL∙s^−1^ using a peristaltic pump connected to one of the two ureters. Although it was possible to use larger volumes, doing so would have required a larger pressurized tank, besides, the chosen volume was sufficient to evaluate catheter performances.

### Testing of standard of care catheters

The ex-vivo porcine LUT model was tested using SOC catheters. Three commercially available male catheter brands were deployed, hereby referred to as “Brand A”, “Brand B”, and “Brand C”. All catheters were of size 12 Charrière (CH12), and with 2 eyelets. The ex-vivo porcine LUT model was set up as described above, filling the pressurized tank with 150 mM NaCl, and the bladder with approximately 205 mL of 150 mM NaCl. Each catheter was tested 5 times in the same bladder and the test was repeated in 3 different fresh male porcine LUTs. This was done to ensure that the biological variance between different porcine LUTs was considered. An “abdominal” pressure of 50 cmH_2_O (i.e., ≈49.0 mbar) was used.

During IC, users are normally instructed to insert the catheter until flow starts and then a bit more, but this varies depending on the specific “Instructions For Use” (IFU) for a specific catheter brand. Catheterizations in the ex-vivo porcine LUT model were performed maintaining a 1 cm gap between the simulated bladder sphincter and the lower edge of the eyelet furthest away from the catheter tip. The catheters were inserted through the urethra and into the bladder and held in position during voiding. The volume emptied from the bladder was progressively collected in a bucket placed on top of a scale. The variation in weight was recorded overtime to allow for the flowrate and for the residual volume at first flow-stop to be calculated. The catheter was held throughout the test by the operator to register tactile sensations. Two tactile feedbacks were recorded by the operator; the sudden pulse of the catheter during flow-stops described in the literature as mucosal suction^21,25^ but also during catheter repositioning, and the rapid pulsation/vibration of the catheter (hammering). Repositioning of the catheter was performed when flow stopped, and until no more liquid was being emptied. Lastly, the catheter was withdrawn, and the test repeated.

The catheter was held during the whole catheterization in the ex-vivo porcine LUT model to ensure for tactile sensations to be felt.

Flowrates were calculated during the first 5 s of voiding. This time interval was chosen because the flowrate remained stable across all catheters tested within it. The residual volume was calculated as the difference between the total volume in the porcine bladder and the emptied volume until the first flow-stop.

### Endoscopic investigation of SOC catheters in the ex-vivo porcine LUT model

A flexible endoscope (Special-Fiberscope, 3.0 mm × 100 cm, Fiberscopes Series, Karl Storz SE & Co. KG, Tuttlingen, Germany) was used to investigate the folding of the bladder around the catheters during catheterization and to visualize the phenomenon of mucosal suction. The endoscope was inserted into the bladder through the urethra, parallel to the catheter, during catheterization. Three male SOC, corresponding to “Brand A”, “Brand B”, and “Brand C” of size CH12 were deployed.

In addition, an endoscope investigation was performed by inserting the endoscope into the catheter lumen to visualize the mucosal suction phenomenon from inside the catheters during catheterization in the ex-vivo porcine LUT model. For the intra-catheter videos, SOC catheters “Brand A”, and “Brand B” CH16, and Brand C of size CH18 were used, to allow for the insertion of the endoscope.

### Impact of the “abdominal” pressure on the performance of SOC catheters

A test was performed to evaluate the impact of testing catheters in the ex-vivo porcine LUT model deploying a lower “abdominal pressure”. SOC catheters (Brands A, B, and C) were tested at 20 and 50 cmH_2_O (i.e., ≈19.6, and ≈49.0 mbar). The two abdominal pressures were chosen to represent a standing and sitting position for a catheter user. The ex-vivo porcine LUT model was prepared as described above. Tactile sensations during catheterization, as well as the residual volume at first flow-stop and the flowrates during the first 5 s of emptying were measured and compared. Each Brand was tested at least 5 times in each bladder. The test was performed in three porcine LUTs deploying a new catheter each time to account for biological variance.

### Intra-catheter pressure measurement

To better understand the phenomena of mucosal suction and hammering, a fiber optic pressure sensor (FISO-LS Fiber Optic Pressure Catheter, model 75-0706, FISO Technologies Inc, Quebec, Canada) was used to measure the pressure variations during catheterization directly inside the catheters. An Evo Chassis (FISO Technologies Inc, Quebec, Canada) was used to power the FISO pressure sensor and constituted the digital interface for data transfer. A custom-made 3D-printed adaptor used to fixate the sensor inside the catheter during testing was fabricated on site (Vero material, J750, Stratasys, Minnesota, USA). The pressure sensor consisted of 0.3-mm wide pressure sensor, recording the pressure variations within a range of + /− 300 mmHg, with a sampling rate of 1000 Hz. The presence of the pressure sensor inside the CH12 SOC catheters caused a reduction in the flowrate of approximately 14%. The pressure sensor was inserted into the catheter keeping a distance of 0.5 cm between the sensor and the lowest edge of the most proximal catheter eyelet. The pressure sensor was held in place by the adaptor throughout the whole catheterization.

Pressure variations were measured in CH12 SOC catheters (Brands A, B, and C) at both 20 and 50 cmH_2_O of “abdominal pressure”. Each catheter was tested at least 5 times in three porcine LUTs to account for biological variance.

### In-vivo animal study

To further validate the biological relevance of the ex-vivo porcine LUT model, animal studies were performed in two female pigs (Landrace x Yorkshire, mix) of 45 kg obtained from a pig herd with the highest health status according to the Danish Specific Pathogen Free system^26^. The animals were sedated according to Stærk et al^27^. In short, the pigs were pre-medicated with medetomidine (Cepetor 0.12 mg∙kg^−1^), butorphanol (Butomidor 0.2 mg∙kg^−1^), and Midazolam (Midazolam 0.1 mg∙kg^−1^). Anaesthesia was induced and maintained on propofol to visualize mucosal suction events, one animal was placed in supine position and catheterized with the SOC catheter Brand A, CH16. The catheter eyelets were visualized by placing a fiber optic endoscope (HOPKINS Forward-oblique Telescope 30°, KARL STORZ, Germany) with protective sheath (Ø = 3.5 mm) inside the catheter. A 50 mL syringe was used to empty the bladder through the working channel of the telescope at a steady flowrate (approximately 6 mL·s^−1^) reflecting a natural bladder emptying. Videos and images were recorded using a TELE PACK VET X LED (KARL STORZ, Germany). Another animal was catheterized using a CH12 Brand A catheter with a mounted pressure sensor to investigate flowrate, mucosal suction pressure, and hammering. The bladder was emptied completely and filled with 200 mL saline. During emptying, the tactile sensation perceived by the operator, flowrate and in-catheter pressure were measured as per the ex-vivo porcine LUT model. Animal experiments were carried out according to the ARRIVE guidelines, EU directive 2010/63/EU for animal experiments, and were approved by the Danish Animal Experiments Inspectorate, license number 2019-15-0201-01,626.

### Statistical analysis

Statistical analysis was performed using GraphPad PRISM (version 9.1.2). T-tests were conducted to compare the results between SOC catheters. All data was considered parametric and Welch corrections were performed when the variances resulted significantly different. No statistical analysis was performed for the qualitative results, such as mucosal suction and hammering. Throughout the article, p-values in the figures are indicated as follows: ns = not significantly different, *p < 0.05, **p < 0.01, ***p < 0.001, and ****p < 0.0001.

## Results and discussion

### Performance of standard of care catheters in the ex-vivo porcine LUT model

**Figure 4:**
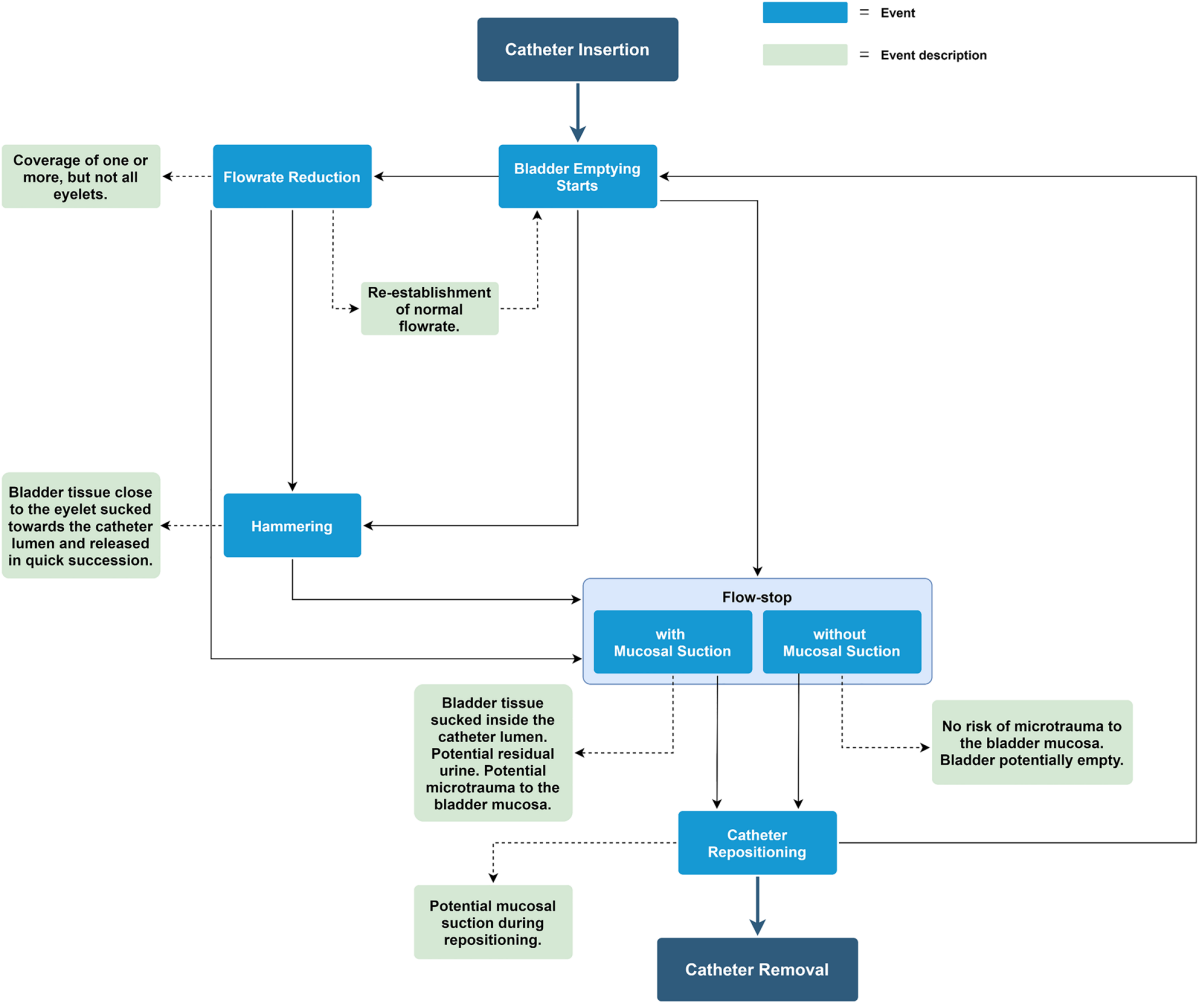
Workflow of an IC. The figure shows the possible events during intermittent catheterization.

During intermittent urinary catheterization (IC), several parameters can be measured to assess the performance of catheters. A standard IC procedure consists of various steps, including opening of the package containing the catheter, potential lubrication of the catheter, insertion of the catheter in the urethra and entrance into bladder. Once the catheter is inserted in the bladder, urine starts flowing with a flowrate which depends on several factors, including the diameter of the catheter´s lumen, the size of the catheter eyelets, and the abdominal pressure. When urine flow stops, IC users are instructed to reposition the catheter until no more urine comes out^8^. Lastly, the catheter is withdrawn from the bladder and the urethra. Figure 4 depicts a simplified description of the events and their sequencing.

Four phenomena were evaluated in the ex-vivo porcine LUT model: flowrate, residual volume, mucosal suction, and hammering. Flowrate corresponds to the speed of the flow during emptying, the remaining volume in the bladder at first flow-stop represents the residual volume, mucosal suction occurs when the bladder mucosa is suddenly sucked inside the catheter lumen stopping the flow, which raises the concern for microtrauma. This event has been previously associated with indwelling catheters^25^, but according to the authors, never in IC. In the ex-vivo porcine LUT model, mucosal suction can be perceived by the operator holding the catheter while performing the catheterization. The tactile perception is felt as a pulse or pulling sensation in the catheter. Lastly, hammering, characterized by a vibration perceived by the operator holding the catheter during voiding. Hammering is not necessarily linked to a flow-stop and can last several seconds if the catheter is not moved.

#### Flowrate in SOC

**Figure 5:**
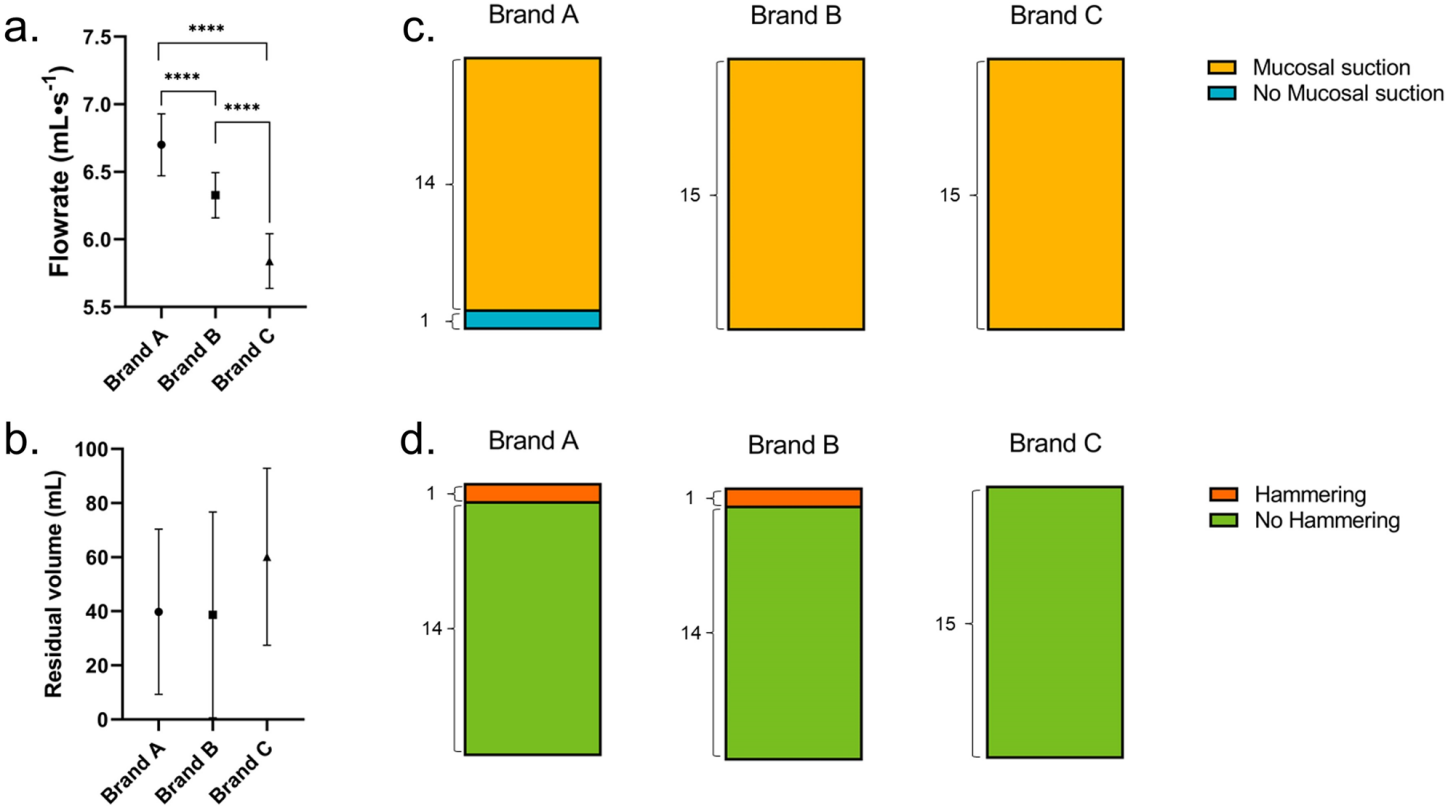
Performance of three standard of care catheters: (**a**) flowrate (mL·s^-1^), calculated in the first 5 s of voiding, (**b**) residual volume (mL), calculated as the difference between the total volume in the porcine bladder and the volume emptied at the first flow-stop, (**c**) mucosal suction perceived by the operator during the first flow-stop, and (**d**) hammering perceived by operator during the whole catheterization. For all Brands, a total of 3 catheters were tested, each catheter was used 5 times and a total of 3 porcine LUTs were used to take the biological variation into account. Significant differences were calculated using a t-test with Welch correction when appropriate.

The calculated flowrates for the SOC catheters (i.e., Brands A, B, and C) can be seen in Fig. 5a. Brand A showed the highest flow rate, equal to 6.70 ± 0.23 mL∙s^−1^, followed by Brand B with 6.32 ± 0.17 mL∙s^−1^, and Brand C with 5.84 ± 0.05 mL∙s^−1^. Statistically significant differences were seen among the flowrates of the different catheters. These differences may be attributed to a combination of factors, such as: different inner diameters and different size and position of the eyelets along the catheter. Please refer to the supplementary material for an overview of the catheters´ inner diameter and eyelet size. Despite the high significant difference, the clinical relevance is evaluated to being negligible as IC users would reasonably not perceive the difference.

#### Residual volume at first flow-stop in SOC

The calculated residual volume at first flow-stop (Fig. 5b) shows no statistically significant differences among the three SOC catheters tested. The residual volumes at first flow-stop were equal to 40 ± 30 mL for Brand A, 39 ± 38 mL for Brand B, and 60 ± 33 mL for Brand C. The relatively high standard deviation could be explained by the direction the eyelets of the catheters were facing during voiding in respect to the nearest bladder wall and by slight variations in the bladder collapsing during emptying.

Residual volume at first flow-stop is of particular interest, especially assuming a premature removal of the catheter before complete bladder emptying. Residual volume is in fact described as one of the risk factor for development of UTIs^9^ and should therefore be minimized.

#### Mucosal suction in SOC

Results for the perceived mucosal suction are depicted in Fig. 5c. As previously described, mucosal suction can occur, and therefore be perceived, both at the first flow-stop, and during repositioning. In this paragraph, only the former of the two was taken into consideration. Mucosal suction during repositioning was however observed numerous times for all SOC catheters tested.

Mucosal suction was perceived in all (but one) catheterizations with all catheters. As mucosal suction is perceived by the operator holding the catheter during emptying, dissipation of the pressure wave along the catheter (affected for example by the catheter material) may influence the operator ability in detecting the event. Despite this, mucosal suction was clearly perceivable in all catheters´ brands tested (Figs. 5 and 9). Mucosal suctions´ intensities, however, varied among tests, also within the same SOC catheter brand. As mucosal suction can occur several times during bladder emptying, concerns for potential mucosal suction-induced microtrauma to the bladder mucosa are very relevant. Especially considering that mucosal suction has been previously described for indwelling catheters^25^, linking the event to the formation of suction marks/oedemas. Microtraumas to the bladder moreover represent an additional factor that was linked to an increased risk of developing UTIs^9^ and should therefore be minimized.

#### Hammering in SOC

Another event that can manifest during IC and that is perceived by the operator holding the catheter, is hammering. As described above, hammering is perceived as a quick succession of mucosal suction-like events that can last several seconds.

The occurrence of hammering was similar among the three SOC catheters tested. Brand A and B manifested 1 event of hammering out of 15 tests whereas no hammering events were perceived by the operator when testing Brand C. Results for the perceived mucosal suction are depicted in Fig. 5d.

The effects of hammering to the bladder mucosa are unknown but the results are included here as the phenomenon was never before described in the literature. What physically happens during hammering is also not yet understood, hammering was in fact never visualized during endoscopic investigations. A hypothesis is that the bladder mucosa, during its deflation, reaches an optimal distance to the catheter´s eyelet to be able to flap (or vibrate), occluding the eyelet for very shorts intervals of time. Flow is in fact not blocked during hammering.

### Endoscopic investigation

**Figure 6:**
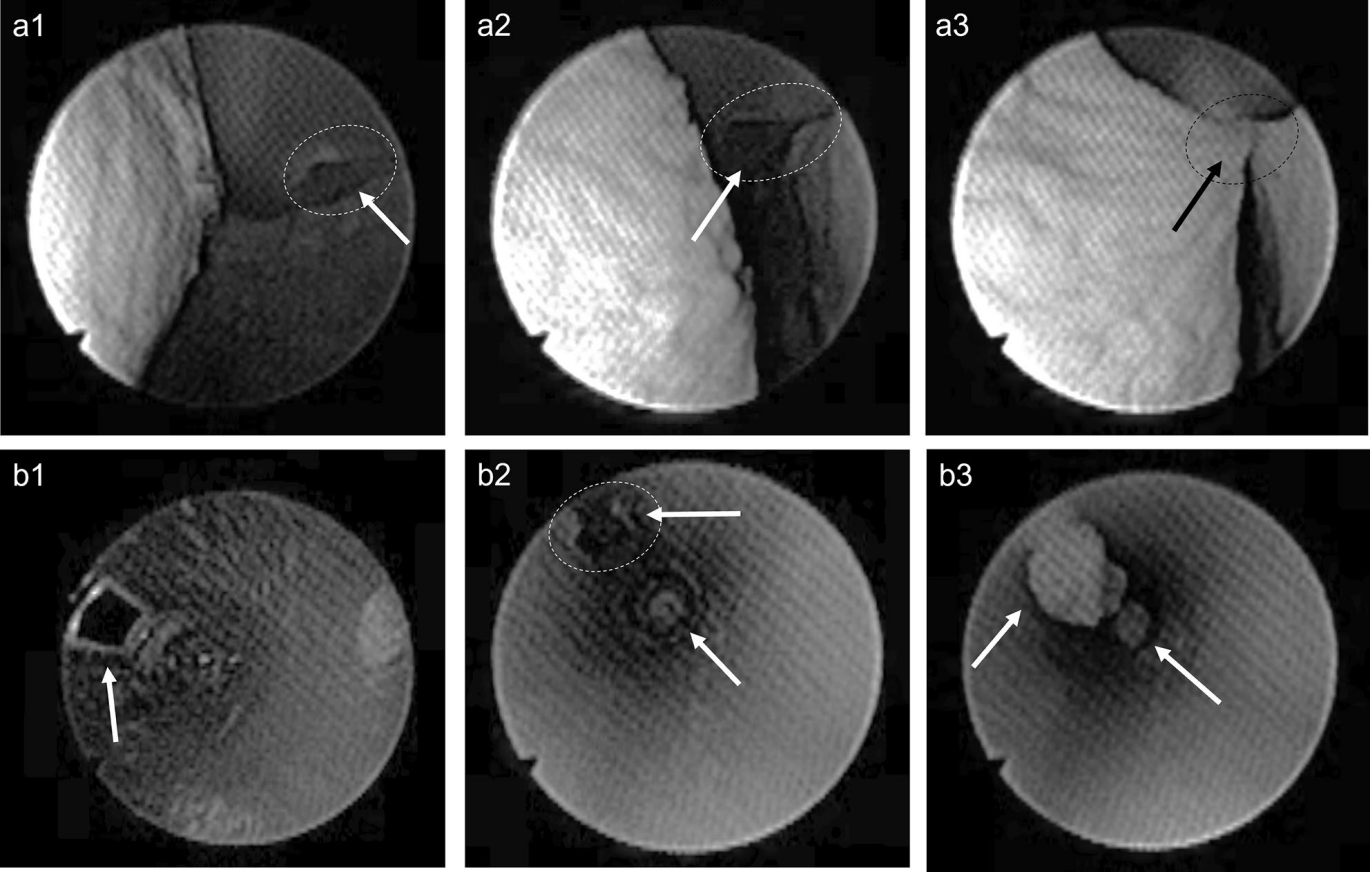
Endoscopic investigation in the ex-vivo porcine LUT model. The (**a**) series depicts the phenomenon of mucosal suction from outside the Brand A catheter (CH12). The (**b**) series depicts, instead, the phenomenon of mucosal suction from inside the Brand A catheter (CH16). (6a1 and 6b1): the eyelet is not blocked, and bladder voiding is continuing. (6a2 and 6b2): the bladder mucosa is approaching the open eyelet, in “6b2” the eyelet further away from the endoscope already shows tissue pulled into the catheter, bladder voiding is continuing. (6a3 and 6b3): Mucosal suction, flow-stop and repositioning is required.

**Figure 7:**
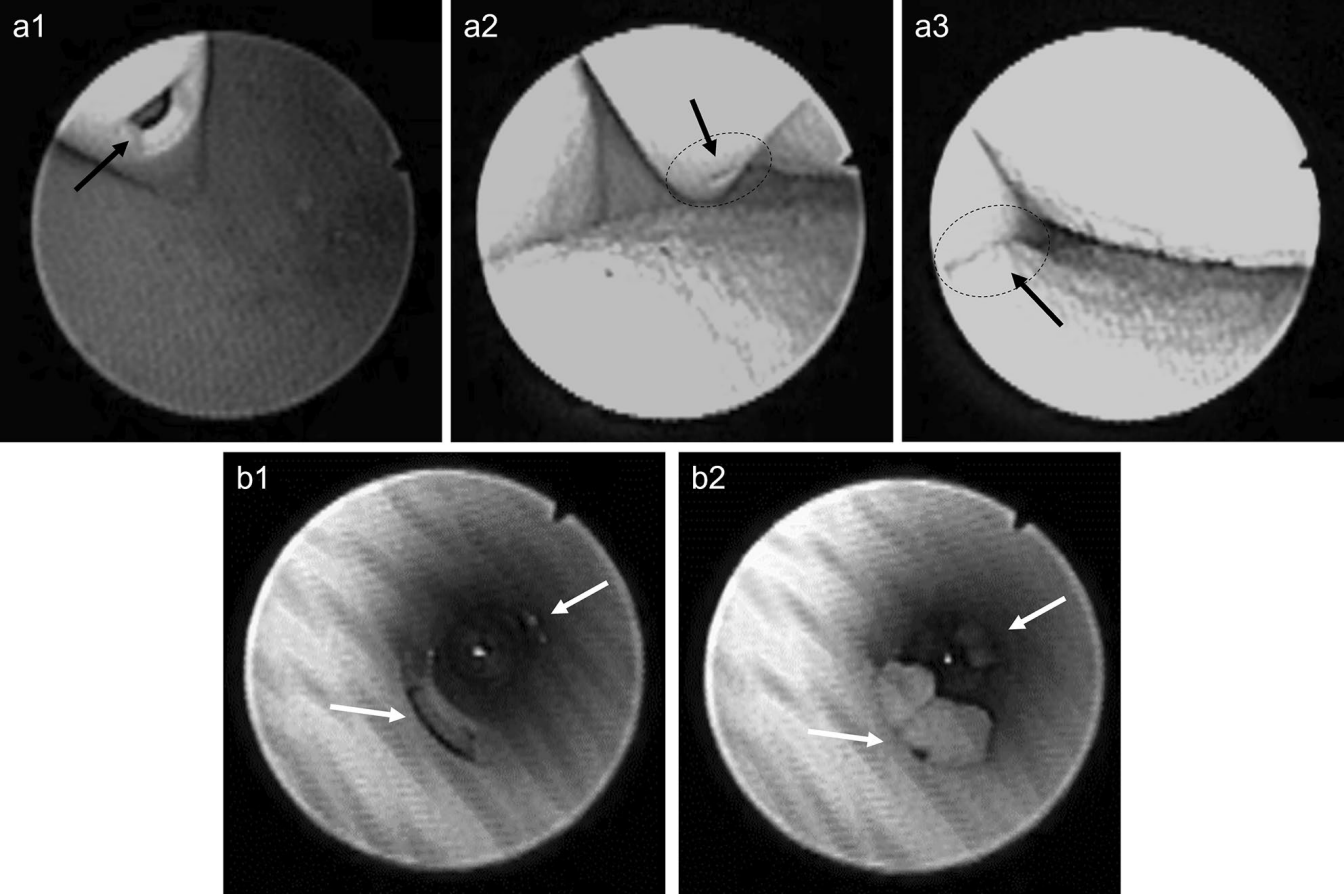
Endoscopic investigation in the ex-vivo porcine LUT model. The (**a**) series depicts the phenomenon of mucosal suction from outside the Brand B catheter (CH12). The (**b**) series depicts, instead, the phenomenon of mucosal suction from inside the Brand B catheter (CH16). Arrows are used in picture 7a2 and 7a3 to guide the reader in visualizing the eyelet. (7a1 and 7b1): the eyelet is visible and bladder emptying is continuing. (7a2): the bladder mucosa is approaching the open eyelet. Flow through the catheter is continuing. (7a3 and 7b2): Mucosal suction, flow-stop, and repositioning is required.

**Figure 8:**
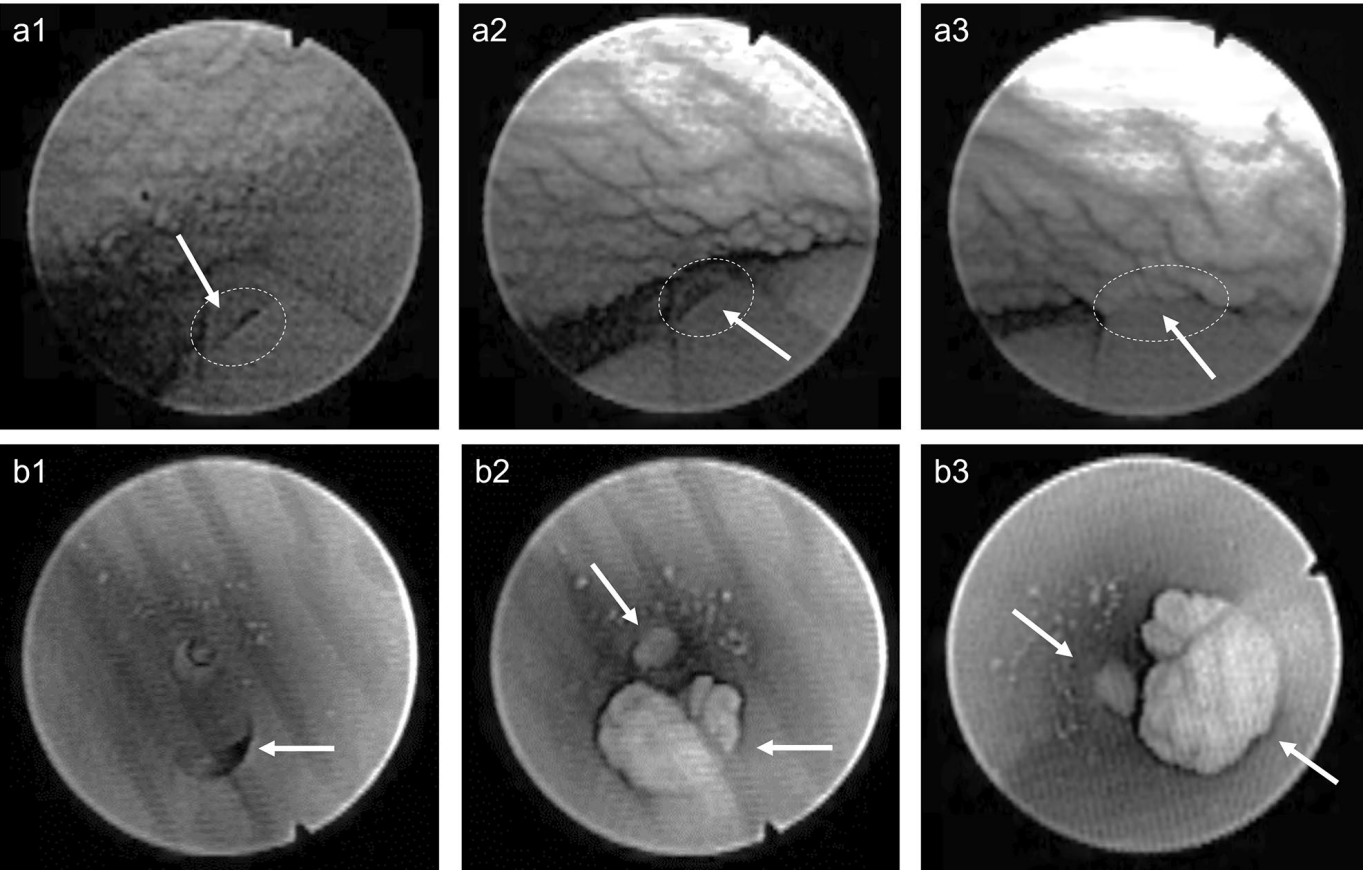
Endoscopic investigation in the ex-vivo porcine LUT model. The (**a**) series depicts the phenomenon of mucosal suction from outside the Brand C catheter (CH12). The (**b**) series depicts, instead, the phenomenon of mucosal suction from inside the Brand C catheter (CH18). (8a1 and 8b1): the eyelet is visible and bladder emptying is continuing. (8a2): the bladder mucosa is approaching the open eyelet, bladder emptying continues. (8a3 and 8b2-3): Mucosal suction, flow-stop and repositioning is required.

To better understand the behavior of urinary catheters in the ex-vivo porcine LUT model, an endoscopic investigation was performed. Two types of endoscopy images were captured, one from the outside of the catheters (Figs. 6, 7, and 8 series “a”) and one from the inside of the catheter (Figs. 6, 7, and 8 series “b”). The former had the advantage of not affecting the flowrate and allowed for a visualization of the bladder mucosa collapsing and folding around the catheter. The latter allowed for a focused visualization of the mucosal suction phenomenon from the inner side of the catheters´ eyelet. Figures 6, 7, and 8 show still images taken from endoscope recordings of SOC catheters Brands A, B, and C, respectively.

Figure 6a1 to a3 presents a series of still images taken from outside a CH12 Brand A catheter in the ex-vivo porcine LUT model. Figure 6 a1 and a2 show one of the eyelets of Brand A with the bladder mucosa closing in towards the catheter, folding around it. In Fig. 6 a3 both sides of the bladder mucosa are observed being sucked into the Brand A eyelet simultaneously.

Figure 6b1 to b3 presents still images of a CH16 Brand A in the ex-vivo porcine LUT model from inside of the catheter. Figure 6 b1 focuses on the lower eyelet (closest to the catheter connector): the eyelet is open and emptying of the bladder in the ex-vivo porcine LUT model is progressing. As emptying continues, the bladder mucosa begins to close in around the catheter, the eyelet further away from the endoscope already shows tissue entering inside the catheter, whilst the closer one remains open (see Fig. 6 b2). A flowrate reduction was associated with this event. Lastly, in Fig. 6 b3 both eyelets are closed, representing an event of mucosal suction seen from inside the catheter. See Videos 1 and 2 in the supporting material for the complete view of the endoscopic investigation.

Similar results were observed for the Brand B and C catheters (see Figs. 7 and 8, respectively). CH12 was deployed for the endoscopic investigation of Brand B and C catheters when the endoscope was kept outside the catheter (see series “a” in Figs. 7 and 8). CH16 and CH18 were used for the intra-catheter endoscopic investigation of Brand B and C respectively. Using CH18 for Brand C was necessary as the endoscope could fit inside a smaller size. See Videos 3, 4, 5, and 6 in the supporting material for a detailed visualization of the whole catheterization with SOC catheters Brand B and C.

It is worth noticing how a considerable amount of tissue was pulled inside the catheters´ lumen even when the endoscope was inserted into the catheter, occupying a large portion of its lumen. The presence of the endoscope determined a significant reduction of the flowrate effectively reducing the pressure variation during mucosal suction. The intensity of mucosal suction, and the volume of tissue visualized in the figures taken from inside the catheter represent consequently an underestimation of the phenomenon. This was true for all SOC catheters tested.

Overall, the endoscopic investigation confirmed the tactile perception of mucosal suction, showing that the phenomenon happens quickly and that it not only happens during the first flow-stop, but also during repositioning. The extent to which the bladder mucosa was sucked into the catheter´s lumen raises the concern for potential microtrauma during IC, even more so considering that 4–6 catheterizations are performed by an IC user daily. Eyelet-related microtrauma due to mucosal suction has been previously reported for indwelling catheters^25^ and it may be argued that similar events could occur with intermittent catheters. At present, there is limited knowledge about the extent of the microtrauma produced by mucosal suction with intermittent catheters. However, the in-vivo animal studies reported later in this work highlight reddening and formation of oedemas.

An additional concerning phenomenon following mucosal suction was observed during the endoscopic investigation: after mucosal suction, catheter repositioning caused scraping of the bladder mucosa. This was clearly visible by both an increased turbidity, and due to the presence of floating agglomerates of tissue (Video 6 in the supporting material). Although this phenomenon may be enhanced by the fact that the model is ex-vivo, the event was confirmed in the in-vivo animal studies and raises an additional concern for microtraumas in SOC catheters for IC users.

### Effect of the abdominal pressure

The abdominal and vesical pressure in humans is subjected to change. One of the parameters affecting this value is the position of patients during measurement. According to the studies conducted by Sullivan et al., and Yao et al^23,24^ the pressure ranges between 20 and 50 cmH_2_O when the subject is standing up, while it ranges between 15 and 40 cmH_2_O when the subject is sitting down. These values are not to be confused with those measured in intensive care units (ICU). In ICU the abdominal pressure is measured with the patient laying down and therefore significantly lower values are expected.

**Figure 9:**
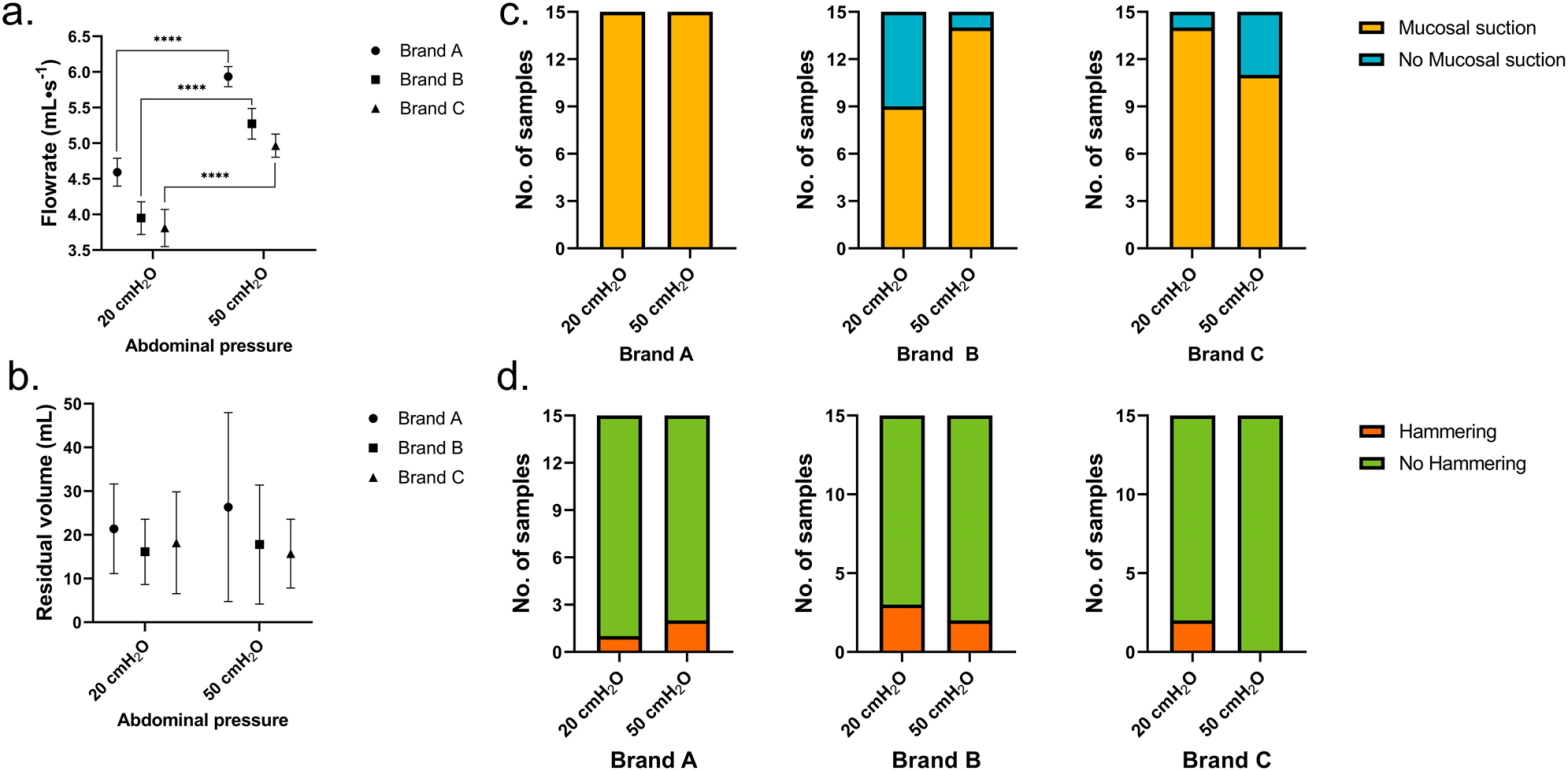
Effect of the abdominal pressure on the performances of three standard of care catheters: (**a**) flowrate (mL·s^–1^), calculated in the first 5 s of voiding, (**b**) residual volume (mL), calculated as the difference between the total volume in the porcine bladder and the volume emptied at the first flow-stop, (**c**) mucosal suction perceived by the operator during the first flow-stop, and (**d**) hammering perceived by operator during the whole catheterization. For all Brands, a total of 3 catheters were tested, each catheter was used 5 times in a total of 3 porcine LUTs to take the biological variation into account (N = 15, SD). The sample size for the flowrate calculation was between 10 and 15 for all brands at both abdominal pressures tested. Significant differences were calculated using a t-test with Welch correction when appropriate.

In this study, two abdominal pressures were tested (20 and 50 cmH_2_O) to mimic a sitting down and standing up position of urinary catheter users respectively. The effect of changing the abdominal pressure was investigated on the flowrate, residual volume at first flow-stop, and occurrence of both mucosal suction and hammering. Results are summarized in Fig. 9. At 20 cmH_2_0, Brand A showed the highest flow rate with 4.59 ± 0.19 mL∙s^−1^, followed by Brand B with 3.95 ± 0.23 mL∙s^−1^ and lastly Brand C with 3.81 ± 0.26 mL∙s^−1^. The same pattern was seen at 50 cmH_2_0 with 5.96 ± 0.16 mL∙s^−1^ for Brand A, followed by Brand B with 5.27 ± 0.22 mL∙s^−1^, and lastly Brand C with 4.97 ± 0.16 mL∙s^−1^. As expected, there was a significant increase in flowrate with increased abdominal pressure.

No significant difference was observed between the residual volume at first flow-stop among the three SOC catheters tested at respectively 20 and 50 cmH_2_0 (Fig. 9b). Brand A had a residual volume of 21 ± 10 mL, Brand B of 16 ± 8 mL, and Brand C 18 ± 12 mL at 20 cmH_2_0. Similarly, at an abdominal pressure of 50 cmH_2_0 brand A had a residual volume of 15 ± 10 mL, Brand B of 18 ± 14 mL, and Brand C 16 ± 8 mL. Smaller residual volumes at first flow-stop were overall measured in this test compared to the results described in paragraph 3.1. This difference supports the need of performing tests in multiple different porcine LUTs to account for the biological variance, standard deviations for the residual volume at first flow-stop are in fact rather large.

No clear pattern was seen comparing the mucosal suction (Fig. 9c) or the hammering (Fig. 9d) at the two different abdominal pressures deployed. Mucosal suction was observed in all catheterizations performed with Brand A. The phenomenon was slightly more frequent at 50 cmH_2_0 for Brand B with 14 events out of 15 catheterizations and only 9 events out 15 at 20 cmH_2_O. For Brand C, the frequency of mucosal suction was higher at the lower abdominal pressure deployed, with 14 out of 15 catheterizations at 20 cmH_2_O, and 11 out of 15 at 50 cmH_2_0. Conversely to what presented in the test in paragraph 3.1, SOC catheters B and C had a lower frequency of mucosal suction at 50 cmH_2_O, especially Brand C.

Hammering was present in nearly all Brands and with no clear pattern. Brand A had 1 hammering event at 20 cmH_2_0 (N = 15) and 2 at 50 cmH_2_0 (N = 15), Brand B had 3 events at 20 cmH_2_0 (N = 15) and 2 at 50 cmH_2_0 (N = 15), and lastly Brand C had 2 events at 20 cmH_2_0 (N = 15) but 0 events at 50 cmH_2_0 (N = 15). Aside for Brand A, hammering was more present than in the previously reported results (Fig. 5).

### Intra-catheter pressure

**Figure 10:**
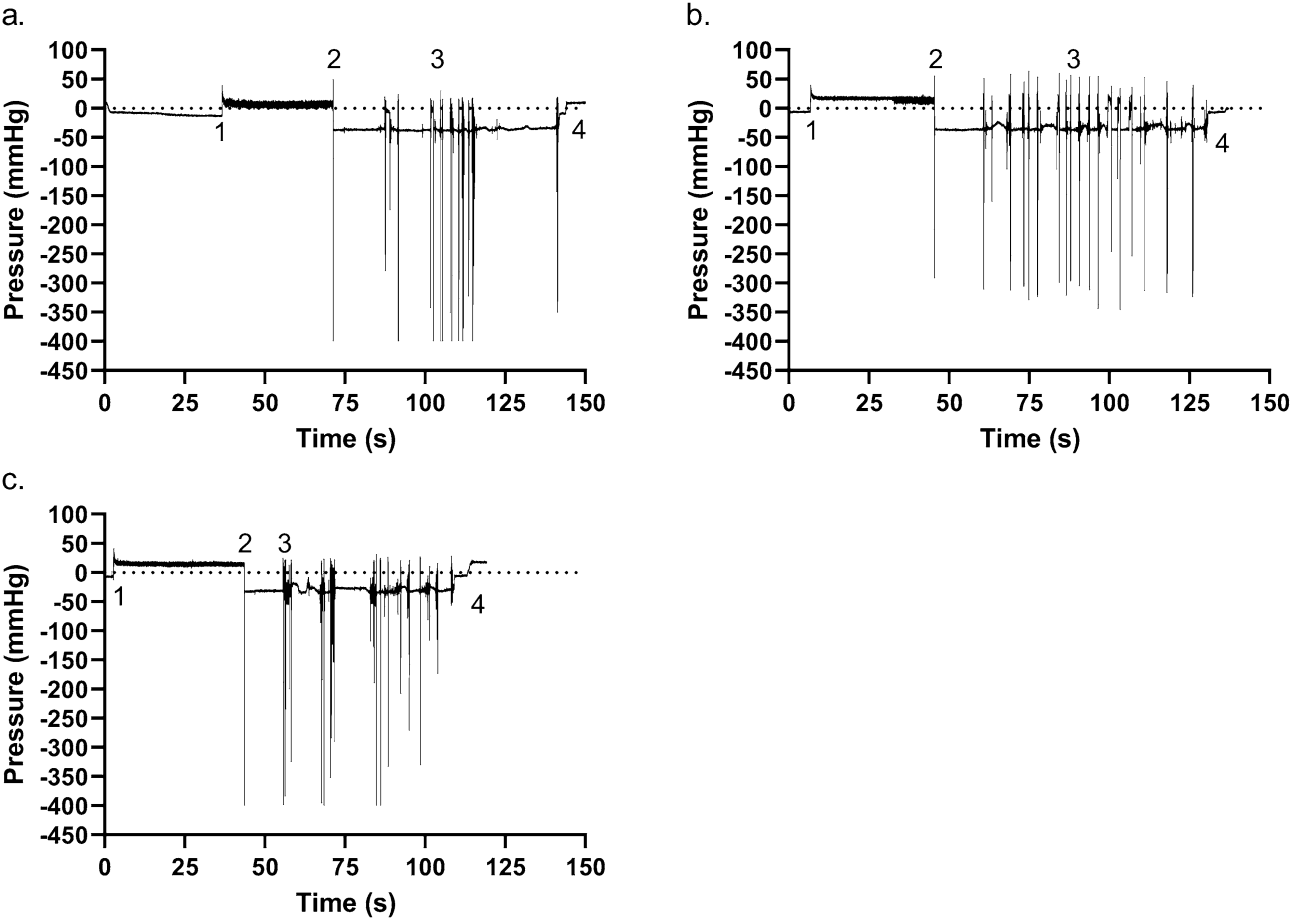
Examples of intra-catheter pressure sensor measurements. (**a**) Brand A, (**b**) Brand B, and (**c**) Brand C. The numbers on the figures represent specific events during IC: (1) insertion of the catheter through the sphincter and into the bladder, emptying starts; (2) flow-stop with an associated mucosal suction; (3) series of mucosal suction events during repositioning; (4) withdrawal of the catheter out of the bladder. The first mucosal suction pressure drop for each example, as indicated by the numbers “2” is zoomed in next to the pressure profile. In the zoomed in picture, the measured profile is shown in blue whereas a gaussian fitting is depicted in red. Brand A, B, and C were tested 5 times in 3 different porcine LUTs (N = 15, SD). An abdominal pressure of 50 cmH_2_O was used.

Throughout the study, the perceivable sensation of mucosal suction, and its intensity, were linked to sudden variations in the pressure in the catheter. To investigate the actual relationship between the two, an intra-catheter pressure analysis was performed. The test was conducted in Brand A, B, and C catheters (Fig. 10).

Similarly, hammering was investigated for a link to a specific pattern in the intra-catheter pressure profiles that could explain the tactile sensation perceived by the operator (Fig. 13).

**Figure 11:**
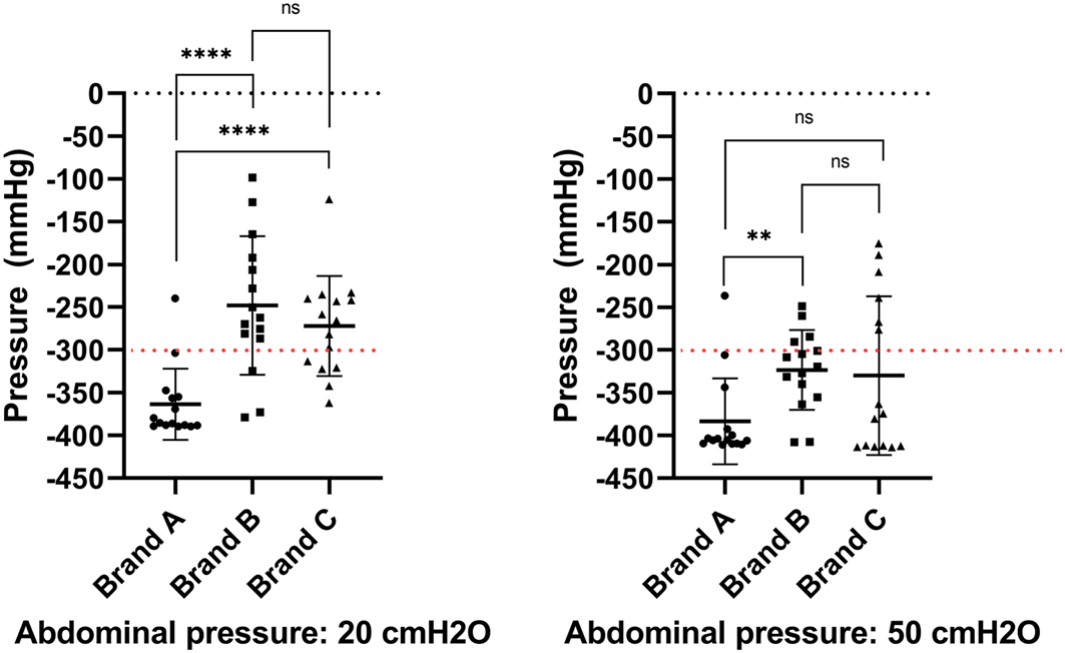
Pressures measured with the intra-catheter pressure sensor at first flow-stop. The test was performed at both 20 and 50 cmH_2_O of abdominal pressure. Each Brand was tested 5 times in 3 different porcine LUTs. The same porcine LUTs where used at both abdominal pressures. Results are reported as individual values, mean and standard deviation. Statistical analysis was performed by means of t-test using Welch’s correction when appropriate.

Pressure variations at first flow-stop were used to compare results among SOC catheters. The analysis was performed both with an abdominal pressure of 20 and 50 cmH_2_O. Since the pressure sensor deployed had a range of ± 300 mmHg (≈ ± 400 mbar), values outside this range could not be measured and all results equal to or more negative than − 400 mbar are less accurate. Therefore, the values reported here represent an underestimate of the real pressure during mucosal suction. Results are summarized in Table 1 and in Fig. 11. The average pressure variation for Brand A was − 364 ± 42 mmHg, − 248 ± 81 mmHg for Brand B and − 272 ± 59 mmHg for Brand C at 20 cmH_2_0. When the abdominal pressure was adjusted to 50 cmH_2_0, the average pressure for Brand A was − 383 ± 50 mmHg, − 323 ± 47 mmHg for Brand B and − 330 ± 93 mmHg for Brand C.

**Table 1:** Results from the intra-catheter pressure sensor in the porcine LUT model. ΔPressure was calculated subtracting the measured pressure at first flow-stop to the baseline pressure.

	ΔPressure ± SD (mmHg)	N	ΔPressure ± SD (mmHg)	N
Abdominal pressure (cmH_2_O)	20		50	
Brand A	– 364 ± 42	15	− 383 ± 50	15
Brand B	– 248 ± 81	15	− 323 ± 47	15
Brand C	– 272 ± 59	15	− 330 ± 93	15

Statistically significant differences were measured comparing the pressure results of the catheters tested at both abdominal pressures deployed. Specifically, the peaks deriving from the mucosal suction phenomena of Brand A resulted significantly different (more negative) than those of Brand B and C at 20 cmH_2_O of abdominal pressure. Conversely, no statistically significant differences were seen comparing Brand B and C. When the abdominal pressure was increased to 50 cmH_2_O, differences between Brands were less prominent. In fact, the only statistically significant difference was measured between Brand A and Brand B, where Brand A showed a more negative average pressure drop. Statistically significant differences were also seen comparing the results between the two abdominal pressures tested. Specifically, statistically significant difference (*p* < 0.05) was seen comparing the average pressures of Brand A, and Brand C, at the two abdominal pressures tested, highly significant difference (*p* < 0.01) was seen comparing the average pressures of Brand B at the two abdominal pressures tested (Fig. 13).

Aside for the pressure peaks during flow-stop, pressure peaks during repositioning can also be seen in Fig. 10a, b, and c. Pressure peaks during repositioning do not differ from those occurring during first flow-stop, potentially bearing the same risk of microtrauma to the bladder tissue. These additional mucosal suction could be associated with discomfort during repositioning, a generally needed procedure for intermittent catheterizations.

**Figure 12:**
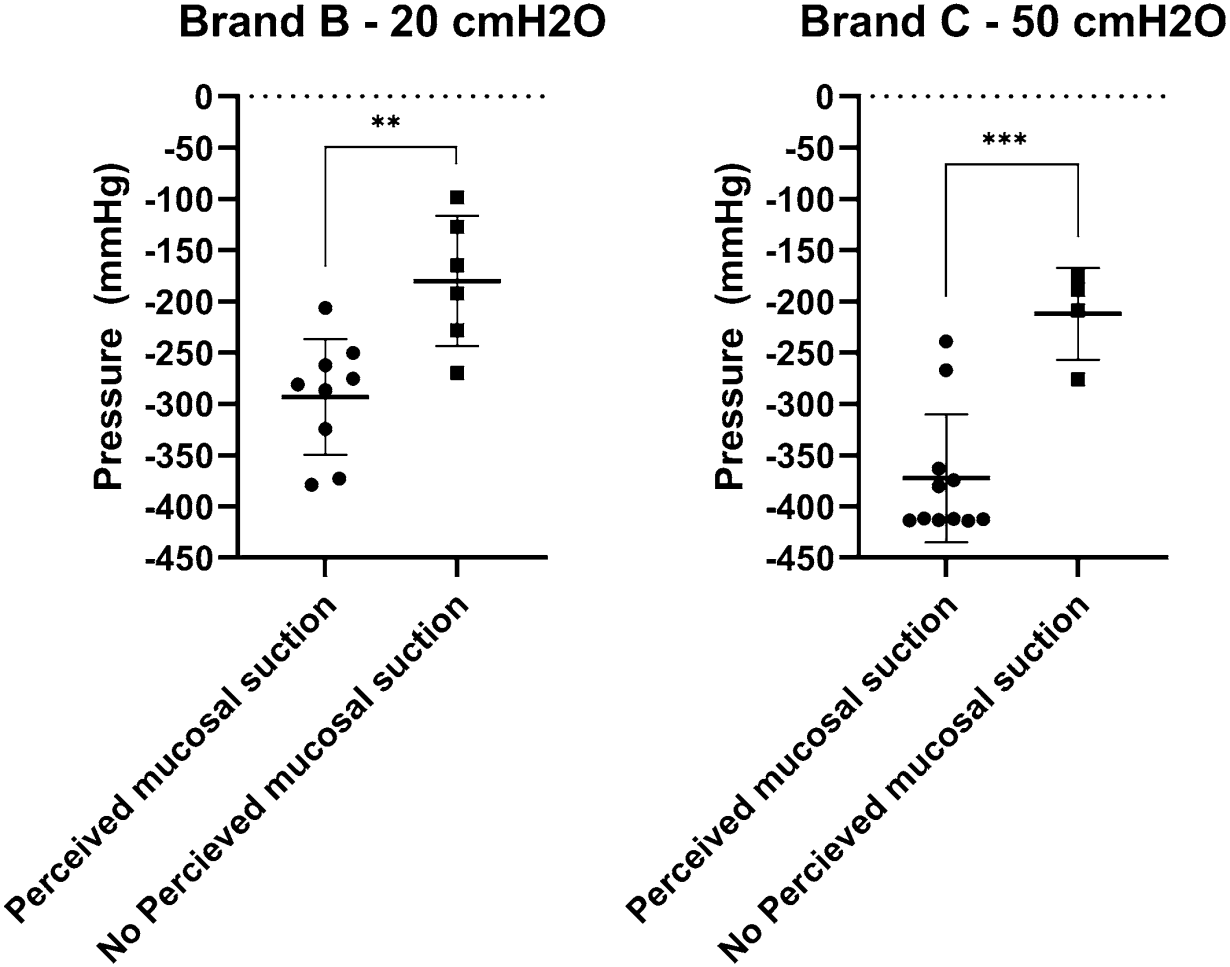
Comparison between the pressure at first flow-stop recorded with the intra-catheter pressure sensor. The results are divided according to whether the mucosal suction phenomenon was perceived by the operator during catheterization, or not. Results are reported as individual values (N = 15, SD). Statistical analysis was performed by means of t-test using Welch’s correction when appropriate.

As previously described, mucosal suction is a phenomenon that is perceived by the operator holding the catheter during emptying in the ex-vivo porcine LUT model. Since the intensity of the phenomenon varies, it is possible that some mucosal suction events are happening but are not being perceived by the operator. The pressure at first flow-stop measured by means of the intra-catheter pressure sensor was cross analyzed with the operator perception of mucosal suction. The results, reported in Fig. 12, show that both at 20 and 50 cmH_2_O of abdominal pressure there was a significant difference in the measured pressure variation at first flow-stop between the cases associated with operator-perceived mucosal suction and those where mucosal suction was instead not perceived by the operator. The measured intra-catheter pressure variation for Brand B at 20 cmH_2_O was equal to − 296 ± 56 mmHg (N = 9, SD) for the tests where mucosal suction was perceived by the operator. Conversely, the intra-catheter pressure variation that could be measured at the first flow-stop for Brand B at 20 cmH_2_O when mucosal suction was not detected by the operator was equal to − 180 ± 64 mmHg (N = 6, SD). A similar scenario was seen for Brand C at 50 cmH_2_O, where the measured intra-catheter pressure variation was equal to − 373 ± 62 mmHg (N = 11, SD) when mucosal suction was perceived by the operator, and to − 212 ± 45 mmHg (N = 4, SD) when mucosal suction was not perceived by the operator. This comparison could only be done between Brand B at 20 cmH_2_O and Brand C at 50 cmH_2_O as there were not enough cases of flow-stops without perceived mucosal suctions in the other SOC catheters.

Overall, a statistically significant correlation between the magnitude of the pressure variation at first flow-stop and the ability to perceive or not perceive the event by the operator performing the catheterization in the ex-vivo porcine LUT model was demonstrated. What remains to be understood is whether a pressure variation of, for example − 250 mmHg, is sufficient to cause discomfort to the IC users, or even cause microtraumas to the bladder mucosa, and if the speed at which the peak is generated has any relevance.

**Figure 13:**
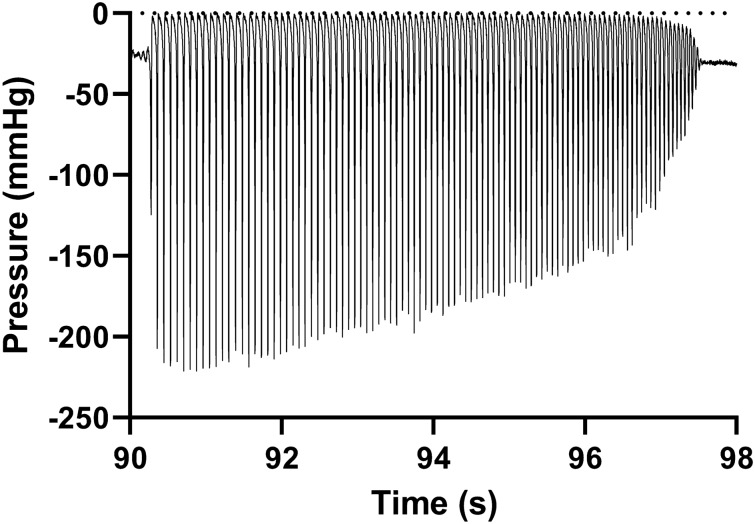
Example of hammering measured with the intra-catheter pressure sensor (Brand C, 20 cmH_2_O).

In addition to the mucosal suction, testing with the intra-catheter pressure sensor in the ex-vivo porcine LUT model allowed for a deeper understanding of the hammering phenomenon. As previously described, hammering is perceived by the operator performing the catheterization in the ex-vivo porcine LUT model as a catheter pulsation/vibration lasting for an extended period of time. An example of this phenomenon, measured with the in-catheter pressure sensor, can be seen in Fig. 13.

The pressure pattern was not identical in every occasion but generally consisted of a series of pressure variations that progressively reduced in amplitude whilst the frequency increased. During the phenomenon, a slower flowrate, equal to about 0.4 mL∙s^−1^, was measured. We hypothesize that during hammering the bladder tissue is very close to the catheter eyelet and mucosal suction is about to happen. However, due to specific conformation of the tissue, or due to the elasticity of that specific portion of the bladder mucosa, the tissue is not immediately pulled inside the catheter, conversely, the tissue vibrates back and forth sequentially closing and re-opening the catheter eyelet. The vibration starts with low frequency and high amplitudes, and as hammering progresses and the energy is dampened, the amplitude decreases with an increase in the frequency. Whether or not this phenomenon is of any clinical relevance remains to be elucidated.

### In-vivo animal studies

To compare how the ex-vivo porcine LUT model reflects catheterization in live animals, a recently established porcine model that facilitates catheterization in-vivo^19,28^ was used. Although the ex-vivo tests were performed on male porcine LUTs, the in-vivo studies had to be performed in female pigs. This was necessary as performing urinary catheterization in male pigs is not possible due to the shape of the penis and the presence of a preputial diverticulum. In the animal studies, only the SOC catheter Brand A was tested. Conversely to humans, and to the ex-vivo porcine LUT model, the flow rate was noticeably slower in the live animals during urinary catheterization. This could be explained by a lower abdominal pressure caused by the supine positioning of the animal and by the lower inclination of the catheter. During catheterization with SOC Brand A in the animal (despite the lower pressure), mucosal suction was perceived by the operator, similarly to what had been found using the ex-vivo porcine LUT model. The phenomenon was perceived several times also during repositioning and by different operators.

**Figure 14:**
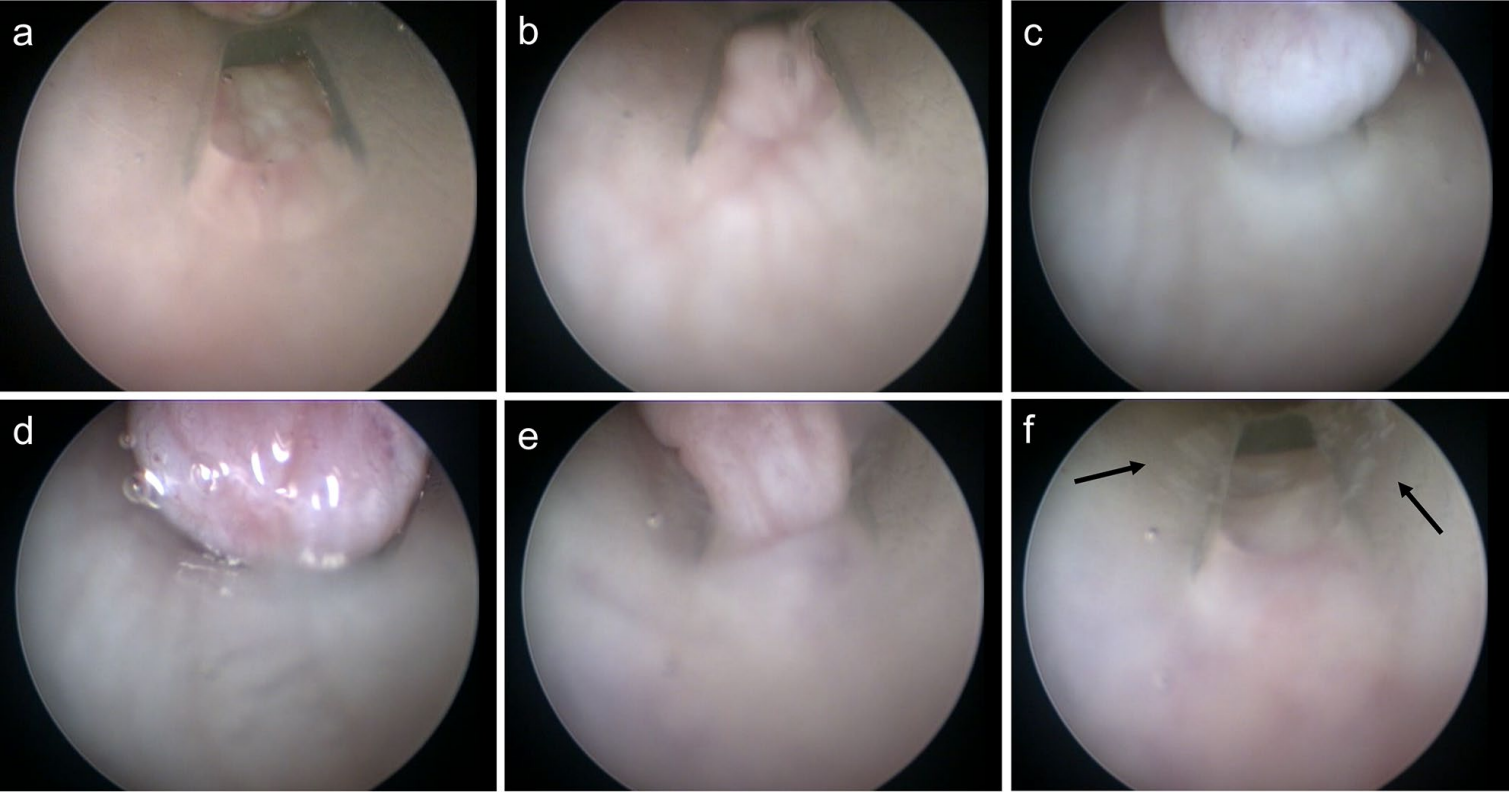
Endoscope investigation in the in-vivo porcine studies. The pictures are snapshot of a video recorded during the catheterization in a pig with a SOC catheter (Brand A, CH16). Tissue from the bladder mucosa progressively closes in towards the catheter eyelet as emptying continues (**a**), the mucosal tissue then begins to be sucked inside the catheter lumen (**b**) when suddenly mucosal suction happens (**c**), the suction increases blood flow on the tissue pulled inside the catheter (**d**), repositioning begins (**e**), after which tissue residues (pointed at with arrows) can be seen on the eyelet sides, probably as a consequence to scraping (**f**).

Endoscopic investigations were performed during catheterization using the Brand A catheter, and the results can be seen in Fig. 14 and Video 7 (supporting material). When emptying the bladder, the mucosal surface was pulled towards the catheter eyelet by the flow of urine (Fig. 14b). Immediately after, the tissue was vigorously sucked inside the catheter lumen (Fig. 14c) after which the catheter was held in position for few seconds leading to increased blood flow in the area and reddening of the tissue (Fig. 14d). When the catheter was repositioned to continue bladder emptying (Fig. 14e), the tissue got pulled out resulting in tissue residues visible at the edge of the SOC catheter eyelet, likely caused by scraping of the tissue during repositioning (Fig. 14f). Occasionally, bleeding was also observed in relation to repositioning (Video 7). These observations support the hypothesis that mucosal suction during IC inflicts microtrauma to the urothelium which likely explains the pinching sensation described by catheter users and may predispose to UTIs^9^. The possibility of inducing microtrauma to the bladder wall during mucosal suction and repositioning of the catheter is of high clinical relevance and should be investigated further. Biopsies of the bladder were unfortunately not performed in this study, as this was outside the scope of this work. A focused investigation will be performed in a following study.

The ex-vivo porcine LUT model uses dead tissue and hence, the increase in blood flow and bleeding events observed in live animals were not detectable. However, the mucosal suction in live pigs generally happened similarly to what was seen in the ex-vivo porcine LUT model, suggesting that the ex-vivo porcine LUT model reflects this event and support its relevance as laboratory model for evaluating catheter performance. It is important to highlight that bladder emptying in the in-vivo studies, when the cystoscope was deployed, was performed through the cystoscope working channel, this might have influenced the pressure gradient created compared to a natural catheter-assisted voiding.

**Figure 15:**
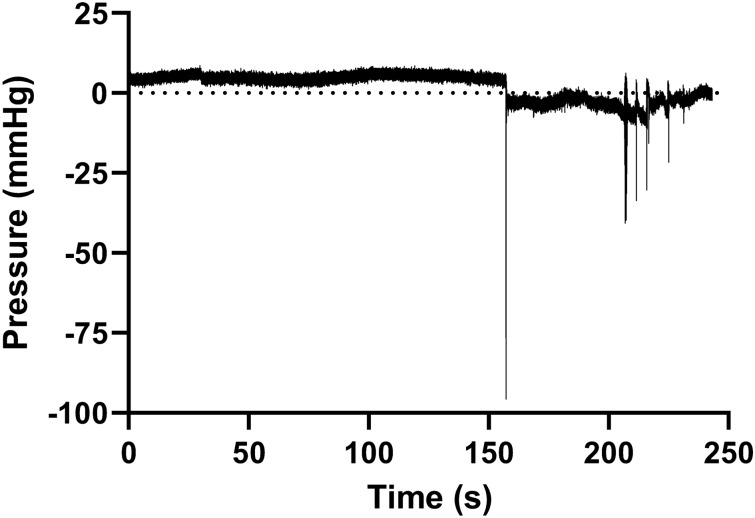
In-vivo in-catheter pressure analysis. The pressure drop visible after the 150 s mark corresponds to the perceived mucosal suction phenomenon.

The change in pressure resulting from mucosal suction was also tested in-vivo using the intra-catheter pressure sensor, testing the SOC catheter Brand A. During bladder emptying, the pressure difference at first flow-stop was equal to − 96 mmHg (Fig. 15). The pressure difference between the one measured in the live pigs and the one measured in the ex-vivo porcine LUT model was probably due to the lower flow rate visualized in the live pigs. Despite this, the pressure peak was easily identified, similarly to the ex-vivo porcine LUT model, and the shape of the pressure peak and the overall pressure curve during bladder emptying reflected those recorded in the ex-vivo porcine LUT model for SOC catheters. Only 1 measurement was obtained in the in-vivo studies. Mucosal suction events and hammering could also be perceived by the operator holding the catheter during voiding in the live pigs.

## Conclusions

In this study, an ex-vivo porcine lower urinary tract model was developed. The model allowed for the evaluation of the performance of SOC intermittent catheters throughout a whole catheterization cycle. It was possible to measure the flowrate during catheter-aided bladder emptying, and to identify flow-stops. It was also possible to calculate the residual volume left in the porcine bladder after flow-stop, residual volume which represents a known risk factor for developing UTIs. By testing with the ex-vivo porcine LUT model, mucosal suction was explored. The event, caused by the sudden pressure variation within the urinary catheter during flow-stop, was associated with a considerable amount of bladder mucosal tissue being quickly pulled inside the catheter through the eyelets. Mucosal suction is concerning as it bears the potential of inducing microtraumas to the bladder mucosa (as shown in the in-vivo animal studies) as well as discomfort to the users of intermittent catheters. The risk of microtrauma may further increase during the repositioning procedure after mucosal suction, when the tissue is tightly held by the suction force. An additional phenomenon was identified during testing, both in the ex-vivo porcine LUT model and in the in-vivo animal study, hereby defined as hammering. This phenomenon has never been previously investigated in intermittent urinary catheters. However, the clinical relevance of this phenomenon is not yet fully understood and requires further investigation.

Overall, the developed model allowed to measure differences among standard of care intermittent catheters available on the market, as well as to understand the effect of abdominal pressure at levels corresponding to those present in patients. The validity of the results obtained in the ex-vivo porcine LUT model were confirmed through testing the same SOC catheters in-vivo in pigs where mucosal suction could also be detected and visualized. The gathered results support the usage of the developed ex-vivo porcine LUT model as an appropriate and relevant method to investigate catheters` performance. Moreover, the results shown in this work support the need for improving the performances of currently available urinary catheters. Future perspectives for the developed model include: modifying the current version to allow for the usage of female porcine LUTs, testing continence care devices other than intermittent catheters (e.g. permanent catheters, artificial sphincters, etc.), evaluate the possibility for using the model as a training tool for urodynamic studies for healthcare professionals.

## Supplementary Information

Supplementary Information 1.

Supplementary Video 1.

Supplementary Video 2.

Supplementary Video 3.

Supplementary Video 4.

Supplementary Video 5.

Supplementary Video 6.

Supplementary Video 7.

## Data Availability

Raw data are available upon request to the corresponding author.
