# Author Correction: Development of an ex-vivo porcine lower urinary tract model to evaluate the performance of urinary catheters

**DOI:** 10.1038/s41598-023-32920-x

**Published:** 2023-04-20

**Authors:** Fabio Tentor, Brit Grønholt Schrøder, Simon Nielsen, Lars Schertiger, Kristian Stærk, Thomas Emil Andersen, Per Bagi, Lene Feldskov Nielsen

**Affiliations:** 1 Coloplast A/S, Holtedam 1, 3050 Humlebæk, Denmark; 2 Research Unit of Clinical Microbiology, University of Southern Denmark, J.B. Winsløws Vej 21, 5000 Odense, Denmark; 3 Department of Clinical Microbiology, Odense University Hospital, J.B. Winsløws Vej 21, 5000 Odense, Denmark; 4 Department of Urology, Centre for Cancer and Organ Diseases, Rigshospitalet, Blegdamsvej 9, 2100 København, Denmark

Correction to: *Scientific Reports* 10.1038/s41598-022-21122-6, published online 24 October 2022

The original version of this Article contained a repeated error, where the pressure unit was incorrectly given as ‘mbar’ instead of ‘mmHg’.

In the Results and discussion section, under the subheading ‘Intra-catheter pressure’,

“The average pressure variation for Brand A was − 364 ± 42 mbar, − 248 ± 81 mbar for Brand B and − 272 ± 59 mbar for Brand C at 20 cmH_2_O. When the abdominal pressure was adjusted to 50 cmH_2_O, the average pressure for Brand A was − 383 ± 50 mbar, − 323 ± 47 mbar for Brand B and − 330 ± 93 mbar for Brand C.”

now reads:

“The average pressure variation for Brand A was − 364 ± 42 mmHg, − 248 ± 81 mmHg for Brand B and − 272 ± 59 mmHg for Brand C at 20 cmH_2_O. When the abdominal pressure was adjusted to 50 cmH_2_O, the average pressure for Brand A was − 383 ± 50 mmHg, − 323 ± 47 mmHg for Brand B and − 330 ± 93 mmHg for Brand C.”

Additionally,

“The measured intra-catheter pressure variation for Brand B at 20 cmH_2_O was equal to − 296 ± 56 mbar (N = 9, SD) for the tests where mucosal suction was perceived by the operator. Conversely, the intra-catheter pressure variation that could be measured at the first flow-stop for Brand B at 20 cmH_2_O when mucosal suction was not detected by the operator was equal to − 180 ± 64 mbar (N = 6, SD). A similar scenario was seen for Brand C at 50 cmH_2_O, where the measured intra-catheter pressure variation was equal to − 373 ± 62 mbar (N = 11, SD) when mucosal suction was perceived by the operator, and to − 212 ± 45 mbar (N = 4, SD) when mucosal suction was not perceived by the operator.”

now reads:

“The measured intra-catheter pressure variation for Brand B at 20 cmH_2_O was equal to − 296 ± 56 mmHg (N = 9, SD) for the tests where mucosal suction was perceived by the operator. Conversely, the intra-catheter pressure variation that could be measured at the first flow-stop for Brand B at 20 cmH_2_O when mucosal suction was not detected by the operator was equal to − 180 ± 64 mmHg (N = 6, SD). A similar scenario was seen for Brand C at 50 cmH_2_O, where the measured intra-catheter pressure variation was equal to − 373 ± 62 mmHg (N = 11, SD) when mucosal suction was perceived by the operator, and to − 212 ± 45 mmHg (N = 4, SD) when mucosal suction was not perceived by the operator.”

Furthermore,

“What remains to be understood is whether a pressure variation of, for example − 250 mbar, is sufficient to cause discomfort to the IC users, or even cause microtraumas to the bladder mucosa, and if the speed at which the peak is generated has any relevance.”

now reads:

“What remains to be understood is whether a pressure variation of, for example − 250 mmHg, is sufficient to cause discomfort to the IC users, or even cause microtraumas to the bladder mucosa, and if the speed at which the peak is generated has any relevance.”

Under the subheading ‘In-vivo animal studies’ of the same section,

“During bladder emptying, the pressure difference at first flowstop was equal to − 96 mbar (Fig. 15).”

now reads:

“During bladder emptying, the pressure difference at first flowstop was equal to − 96 mmHg (Fig. 15).”

Finally, the error was also present in Table 1 and in Figures 10, 11, 12, 13 and 15.

The correct and incorrect values of Table 1 appear below.

Table 1

Incorrect:

**Table:** 

	ΔPressure ± SD (mbar)	N	ΔPressure ± SD (mbar)	N

Correct:

**Table:** 

	ΔPressure ± SD (mmHg)	N	ΔPressure ± SD (mmHg)	N

The original Figures 10, 11, 12, 13 and 15, and accompanying legends appear below.

The original Article has been corrected.

**Figure 10:**
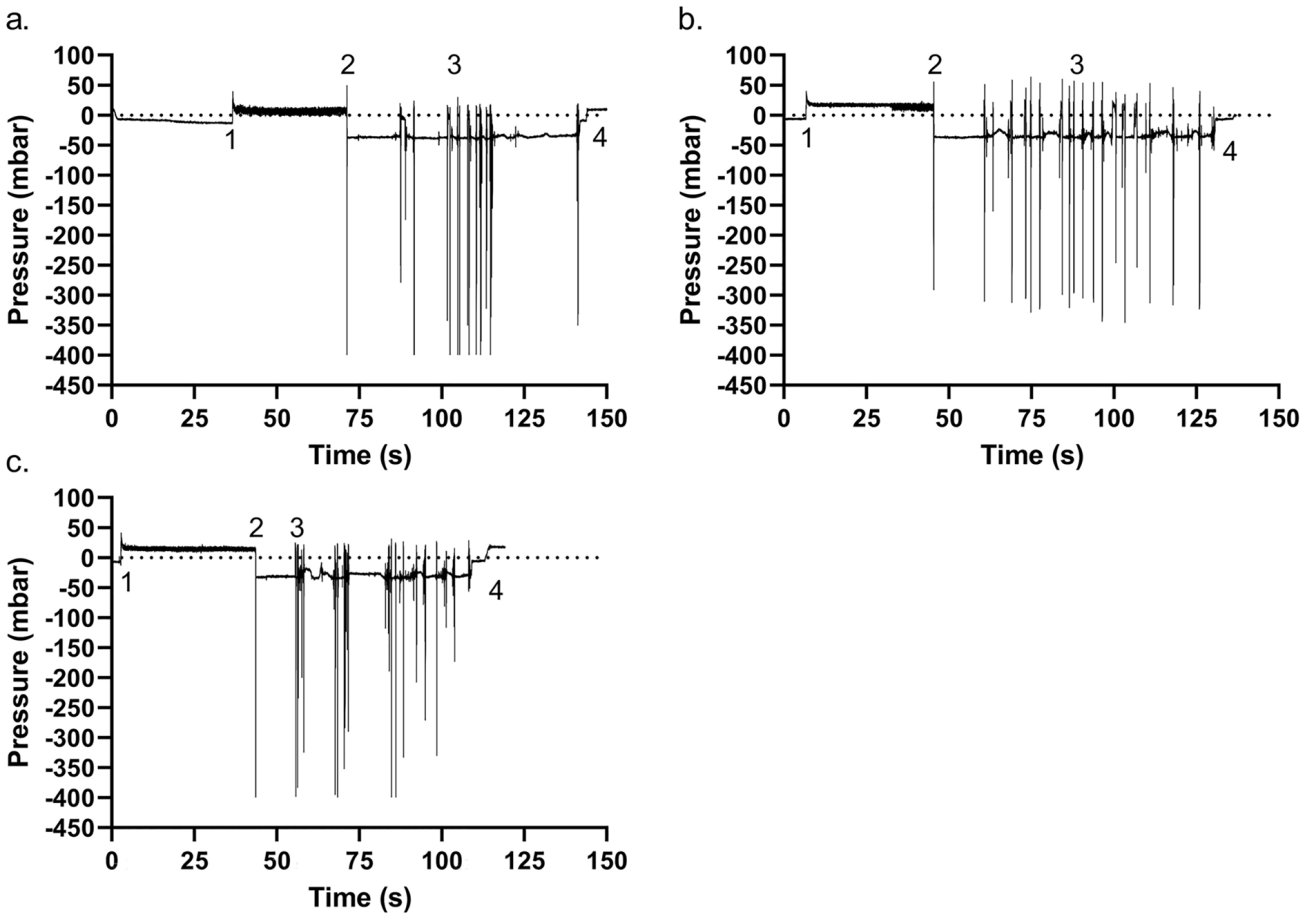
Examples of intra-catheter pressure sensor measurements. (**a**) Brand A, (**b**) Brand B, and (**c**) Brand C. The numbers on the figures represent specific events during IC: (1) insertion of the catheter through the sphincter and into the bladder, emptying starts; (2) flow-stop with an associated mucosal suction; (3) series of mucosal suction events during repositioning; (4) withdrawal of the catheter out of the bladder. The first mucosal suction pressure drop for each example, as indicated by the numbers “2” is zoomed in next to the pressure profile. In the zoomed in picture, the measured profile is shown in blue whereas a gaussian fitting is depicted in red. Brand A, B, and C were tested 5 times in 3 different porcine LUTs (N = 15, SD). An abdominal pressure of 50 cmH_2_O was used.

**Figure 11:**
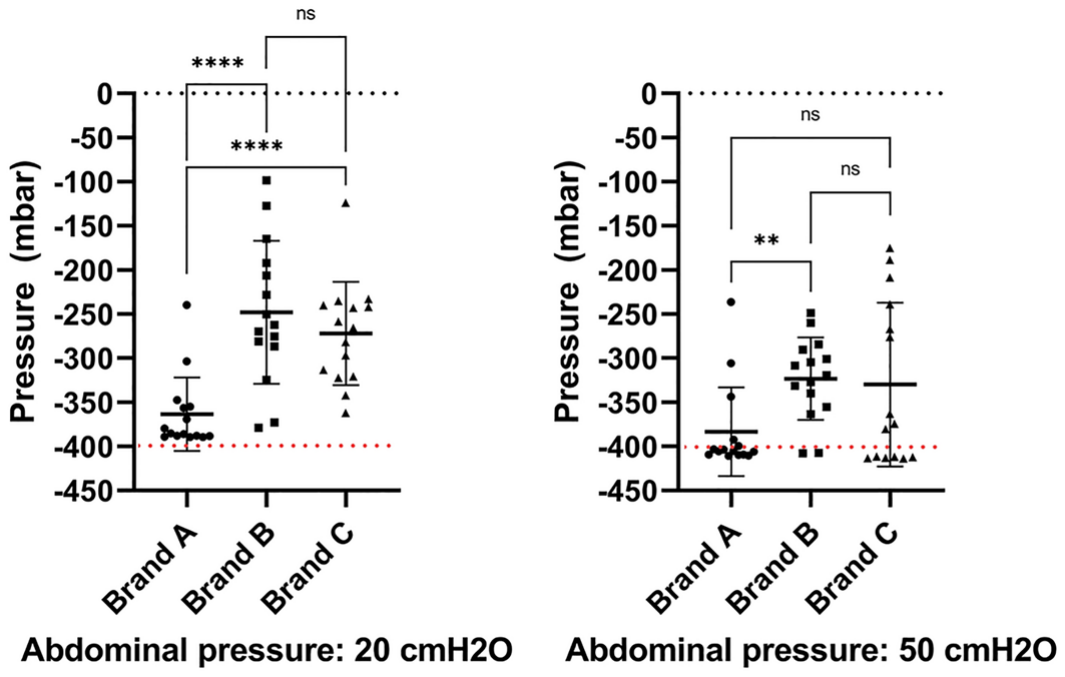
Pressures measured with the intra-catheter pressure sensor at first flow-stop. The test was performed at both 20 and 50 cmH_2_O of abdominal pressure. Each Brand was tested 5 times in 3 different porcine LUTs. The same porcine LUTs where used at both abdominal pressures. Results are reported as individual values, mean and standard deviation. Statistical analysis was performed by means of t-test using Welch´s correction when appropriate.

**Figure 12:**
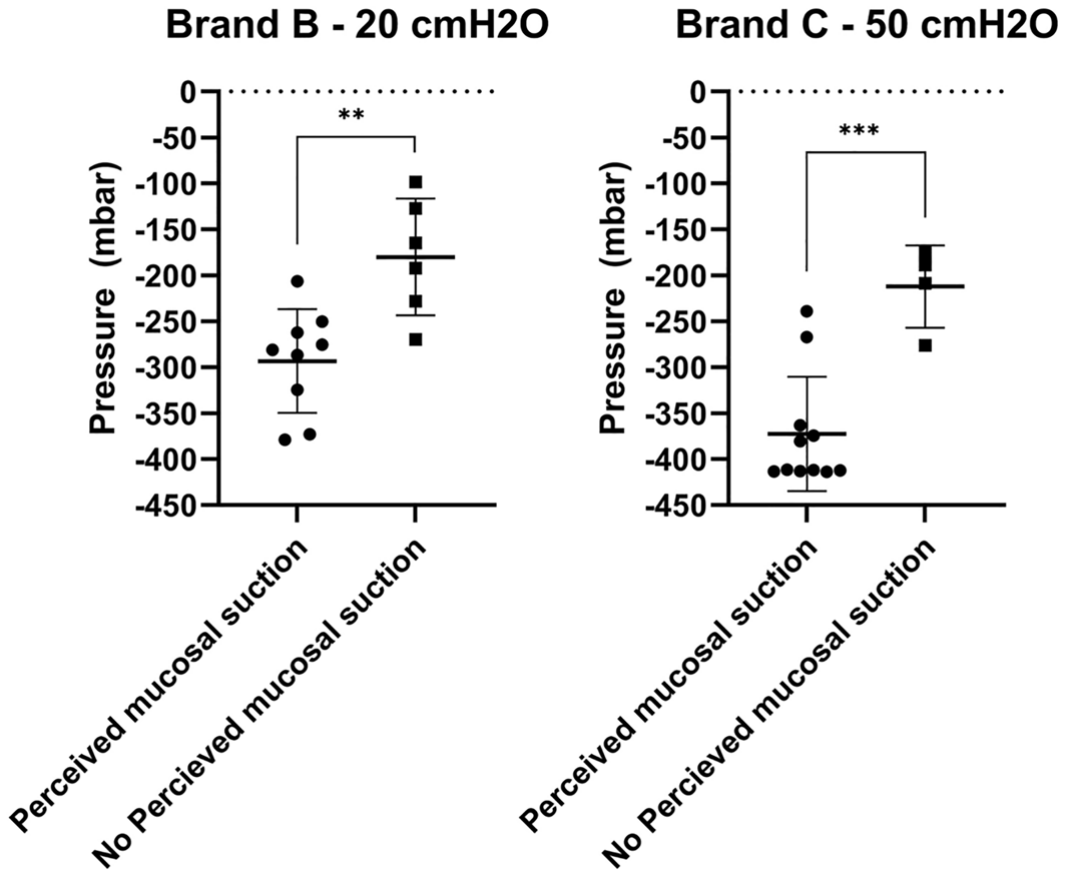
Comparison between the pressure at first flow-stop recorded with the intra-catheter pressure sensor. The results are divided according to whether the mucosal suction phenomenon was perceived by the operator during catheterization, or not. Results are reported as individual values (N = 15, SD). Statistical analysis was performed by means of t-test using Welch´s correction when appropriate.

**Figure 13:**
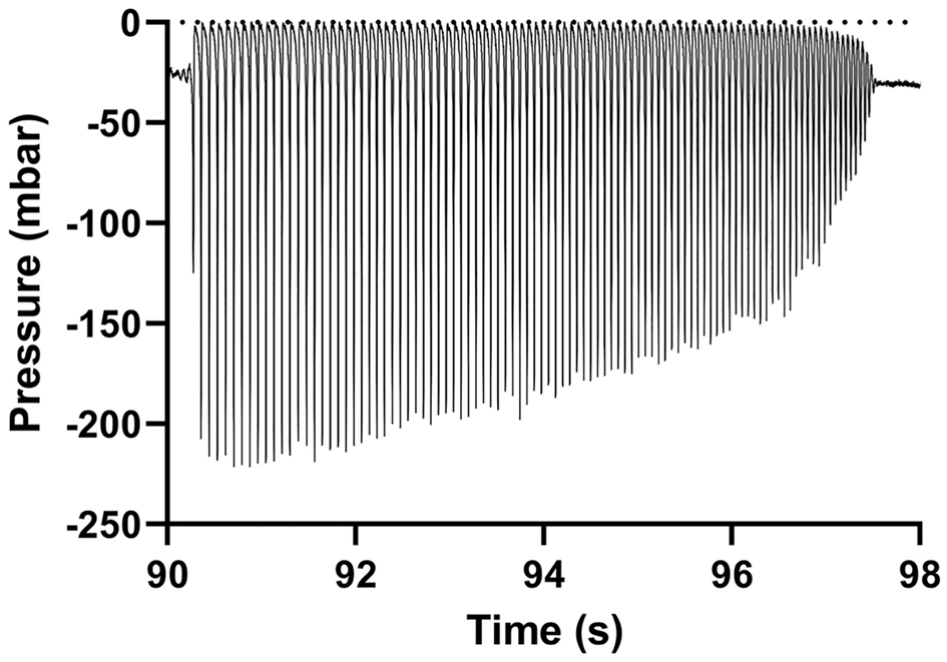
Example of hammering measured with the intra-catheter pressure sensor (Brand C, 20 cmH_2_O).

**Figure 15:**
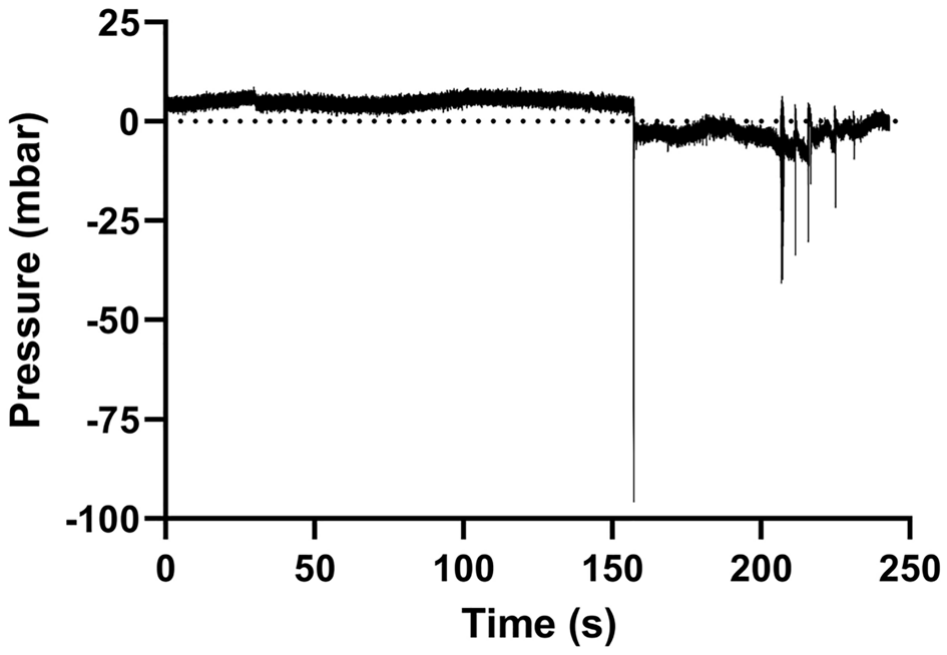
In-vivo in-catheter pressure analysis. The pressure drop visible after the 150 s mark corresponds to the perceived mucosal suction phenomenon.

