# Supplementary material for: Development of an ex-vivo porcine lower urinary tract model to evaluate the performance of urinary catheters
Source: Sci Rep. 2022 Oct 24;12:17818. doi: 10.1038/s41598-022-21122-6 (PMC9592621; doi:10.1038/s41598-022-21122-6)
Supplement: Supplementary file 1 — Supplementary Information 1. [file 41598_2022_21122_MOESM1_ESM.docx]

**Supplementary material for:**

**Development of an *ex-vivo* porcine lower urinary tract model to evaluate the performance of urinary catheters.**

Fabio Tentor*^,a^, Brit Grønholt Schrøder^a^, Simon Nielsen^a^, Lars Schertiger^a^, Kristian Stærk^b,c^, Thomas Emil Andersen^b,c^, Per Bagi^d^, and Lene Feldskov Nielsen^a^

* Corresponding author

^a^ Coloplast A/S, Holtedam 1, Humlebæk, 3050, Denmark

^b^ Research unit of Clinical Microbiology, University of Southern Denmark, J.B. Winsløws Vej 21, Odense, 5000, Denmark

^c^ Department of Clinical Microbiology, Odense University Hospital, J.B. Winsløws Vej 21, Odense, 5000, Denmark

^d^ Department of Urology, Centre for Cancer and Organ Diseases, Rigshospitalet, Blegdamsvej 9, København Ø, 2100, Denmark

**1. Captions for the endoscope videos**

**Video 1.** Endoscopic investigation in the ex-vivo porcine LUT model captured from the outside of a Brand A catheter (CH12).

**Video 2.** Endoscopic investigation in the ex-vivo porcine LUT model captured from the inside of a Brand A catheter (CH16).

**Video 3.** Endoscopic investigation in the ex-vivo porcine LUT model captured from the outside of a Brand B catheter (CH12).

**Video 4.** Endoscopic investigation in the ex-vivo porcine LUT model captured from the Inside of a Brand B catheter (CH16).

**Video 5.** Endoscopic investigation in the ex-vivo porcine LUT model captured from the outside of a Brand C catheter (CH12).

**Video 6.** Endoscopic investigation in the ex-vivo porcine LUT model captured from the Inside of a Brand C catheter (CH18).

**Video 7.** Endoscopic investigation performed in the *in-vivo­* animal study, captured from the Inside of a Brand A catheter (CH16).

**2. Standard of care catheters dimensions**

The dimensions of the three standard of care catheters used were measured by means of a caliper. The diameter of the catheters` lumen, the eyelets` length, and the eyelets` width were measured 5 times and the averages compared by means of an unpaired t-test. Results are summarized in Table 1.

Statistically significant differences were found comparing the dimensions of the three catheters. Looking at the diameter of catheters` lumen, no statistical difference was seen between Brand A and Brand B (p = 0.2). Significant differences were instead seen comparing Brand A with Brand C (p < 0.0001) and Brand B with Brand C (p < 0.0001). Similarly, no significant differences were measured comparing the eyelets` length for Brand A and Brand B (p = 0.1553) whereas significant differences were seen comparing Brand A with Brand C (p = 0.0002) and Brand B with Brand C (p < 0.0001). Lastly, significant differences were measured for the eyelets` widths between all catheters. Brand A had larger eyelets than both Brand B (p < 0.0001) and Brand C (p = 0.0451) while Brand C had larger eyelets than Brand B (p < 0.0001).

**Table 1.** Dimensions of standard of care catheters.

| **Catheter** | **Lumen diameter (mm)** | | | **Eyelet length (mm)** | | | **Eyelet width (mm)** | | |
| --- | --- | --- | --- | --- | --- | --- | --- | --- | --- |
|  | **AVG** | **S.D.** | **N** | **AVG** | **S.D.** | **N** | **AVG** | **S.D.** | **N** |
| **Brand A** | 2.57 | 0.05 | 5 | 4.05 | 0.09 | 5 | 1.60 | 0.04 | 5 |
| **Brand B** | 2.61 | 0.04 | 5 | 4.13 | 0.07 | 5 | 1.39 | 0.05 | 5 |
| **Brand C** | 2.28 | 0.05 | 5 | 3.70 | 0.08 | 5 | 1.66 | 0.04 | 5 |
